# Identification, characterization and quantification of xanthones from *Fridericia formosa* leaves extract with antiviral activity

**DOI:** 10.1038/s41598-024-51881-3

**Published:** 2024-01-26

**Authors:** Luana Beatriz Araújo Vaz, Tatiane Roquete Amparo, Adriana Cotta Cardoso Reis, Breno de Mello Silva, Cíntia Lopes de Brito Magalhães, Markus Kohlhoff, Geraldo Célio Brandão

**Affiliations:** 1 Programa de Pós-Graduação em Ciências Farmacêuticas, Escola de Farmácia, Universidade Federal de Ouro Preto, Campus Morro Do Cruzeiro, Ouro Preto, Minas Gerais Zip Code 35.402-163 Brazil; 2 Departamento de Ciências Biológicas, ICEB, Universidade Federal de Ouro Preto, Campus Morro Do Cruzeiro, Ouro Preto, MG Zip Code 35.402-163 Brazil; 3 Laboratório de Química de Produtos Naturais Bioativos, Fundação Oswaldo Cruz, Instituto René Rachou, Belo Horizonte, Minas Gerais Zip Code 30.190-009 Brazil

**Keywords:** Plant sciences, Medical research

## Abstract

*Fridericia formosa* (Bureau) L.G. Lohmann (Bignonaceae) is a neotropical liana species found in the Cerrado biome in Brazil. It has been of great interest to the scientific community due to its potential as a source of new antivirals, including xanthones derived from mangiferin. In this context, the present study aimed to characterize and quantify the xanthones present in the ethanol extract of this species using high performance liquid chromatography. Additionally, the antiviral activity against Chikungunya, Zika, and Mayaro viruses was evaluated. The chromatographic analyses partially identified twenty-six xanthones, among which only fourteen had already been described in the literature. The xanthones mangiferin, 2′-*O-trans*-caffeoylmangiferin, and 2*′-O-trans*-coumaroylmangiferin, are present in higher quantities in the extract, at concentrations of 9.65%, 10.68%, and 3.41% w/w, respectively. In antiviral assays, the extract inhibited the multiplication cycle only for the Mayaro virus with a CE_50_ of 36.1 μg/mL. Among the isolated xanthones, 2′-*O-trans*-coumaroylmangiferin and 2*′-O-trans*-cinnamoylmangiferin inhibited the viral cytopathic effect with CE_50_ values of 180.6 and 149.4 μg/mL, respectively. Therefore, the extract from *F. formosa* leaves, which has a high content of xanthones, has antiviral potential and can be a source of new mangiferin derivatives.

## Introduction

*Fridericia* (*Arrabidaea*) is the second genus of Bignoniaceae with more than 65 species of plants found mainly in the tropical and subtropical regions of South America^1,2^. These plants have been the subject of growing interest due to their chemical properties and potential medicinal use^3,4^. In the medicinal context, various species of *Fridericia* have traditionally been used by indigenous communities to treat a variety of health conditions. Among the medicinal uses and properties of species in this genus, noteworthy are their anti-inflammatory activity, effectiveness against infections, treatment of gastrointestinal disorders, and renal disorders, among others^5^.

The chemistry of plant species within the genus *Fridericia* is notable due to the presence of chemical compounds such as terpenes, flavonoids, tannins, and other phenolic compounds^3,4^. These components confer bioactive properties to the plants, making them the subject of pharmacological and ethnopharmacological studies. Xanthones were first described as chemical constituents of extracts from species in this genus by Pauletti et al.^6^, with the isolation of mangiferin and five other derivatives from the extract of the species *F. samydoides*. Martin et al.^7^ isolated mangiferin, isomangiferin, and six new derivatives from extracts of the species *F. patellifera*. Recently, Brandão et al. and Fonseca et al.^8,9^ isolated mangiferin and its derivatives from the species *F. formosa* and *F. samydoides,* respectively.

Arboviruses, such as the Chikungunya, Zika virus, dengue virus, and others, pose a global threat due to their spread by vectors and the frequent outbreaks they cause^10^. Often, there are no specific antiviral treatments available, and prevention, mainly through vector control, is the primary method of combat^10^. However, research on natural products has revealed a range of advantages in the search for new anti-arboviral agents^11^. These substances are found in plants, microorganisms, insects, and even marine animals, opening a wide array of options for the discovery of antiviral agents^10,12^.

The interest in the search for antivirals from extracts of species within the *Fridericia* genus has arisen due to the fact that some species are medicinally used for the symptomatic treatment of infections with a possible viral cause^3,4^. The potential antiviral action of products and compounds obtained from species within the *Fridericia* genus has been demonstrated in recent scientific publications^13^ and product patents^14^. Brandão et al.^2,3^ have published works reporting the antiviral action of species within the genus against Herpes simplex virus type 1, Vaccinia WR virus, and Encephalomyocarditis virus. Brandão et al.^8^ also describe the inhibition of the cytopathic effect in cell culture by dengue virus, Herpes simplex virus type 1, and Vaccinia WR virus by extracts and constituents obtained from the species *F. formosa*. Meanwhile, Fonseca et al.^9^ describe the antiviral action of extracts and flavonoids from *F. samydoides*. However, the antiviral activity of *F. formosa* has not yet been evaluated for Chikungunya, Mayaro, and Zika viruses.

Studies of different species within this genus, in which the presence of xanthones has been confirmed through the isolation of major compounds, have shown that in addition to the main compounds, other components of this chemical class are present in small quantities or even in trace amounts in these extracts^9^. Therefore, the use of high-performance liquid chromatography coupled with hyphenated techniques (UV and MS) can contribute to the identification of possible new compounds present in low concentrations. In this context, the present study describes the quantification of mangiferin and cinnamoylated derivatives, as well as the identification and partial characterization of a series of xanthones present in the leaves extract of the species *F. formosa* by LC-DAD-HRMS. Additionally, the potential antiviral effect of the extract, fractions, and isolated xanthones against Chikungunya, Mayaro, and Zika viruses was evaluated.

## Results and discussion

### Characterization of the chemical structure of xanthones by LC-DAD-HRMS

Ethanol, which possesses amphipathic properties, is a potential solvent for extracting various classes of secondary metabolites, including tannins, flavonoids, terpenoids, sterols, alkaloids, and xanthones. Therefore, it is a suitable choice for studies with phytochemical characterization and biological activity screening of natural products^15,16^. Additionally, ethanol is low-toxic, environmentally friendly, and suitable for large-scale processes^17^. Therefore, ethanol was chosen for extraction of *F. formosa* leaves and the LC-DAD-HRMS showed that this extract is rich in xanthones.

Most of the known xanthones are of natural origin, occurring as quite common secondary metabolites in various families of fungi, lichens, and mainly in plants^18,19^. In the present study, chromatographic analyses allowed us to identify the presence of twenty-six xanthones in the ethanolic extract of the leaves of *F. formosa*. The parameters adopted in the analyses included retention time, UV absorption maximum, fragmentation pattern, and exact mass. A maximum error of 5.0 ppm in relation to the calculated exact mass was accepted. However, none of the identified compounds had an error exceeding 2.0 ppm (Table 1). Furthermore, some compounds were identified by comparison with data obtained from isolated authentic samples. All identified compounds exhibited at least four UV absorption maximum in regions characteristic of mangiferin.

**Table 1:** Xanthones partially identified in extract of *Fridericia formosa* by LC-DAD-MS.

	Compound	Molecular Formula	RT (min)	UV (nm)	HRMS [M+H]^+^ (*m/z*)	Error ppm	[M−H]^−^ (*m/z*)	Characteristic *m/z* of Ions in Positive Ion Mode (%)
1	Isomangiferin	C_19_H_18_O_11_	11.0	218, 258, 318, 373	423.0922	1.2	421.23	423.0920 (48.6), 405.0814 (33.6), 357.0604 (20.6), 339.0498 (18.3), 327.0500 (35.5), 303.0497 (73.2), 299.0548 (23.0), 274.0434 (20.0), 273.0392 (100.0), 257.0440 (20.9)
2	Mangiferin	C_19_H_18_O_11_	12.1	190, 218, 258, 318, 373	423.0921	1.2	421.25	423.0922 (46.5), 405.0817 (30.4), 387.0710 (14.6), 339.0500 (15.0), 327.0500 (27.2), 303.0500 (61.0), 299.0551 (18.8), 274.0438 (14.5), 273.0395 (100.0), 257.0446 (19.9)
3	Mangiferin-*O*-pentosyl derivative	C_24_H_26_O_15_	12.6	218, 240, 258, 318, 365	555.1339	2.0	553.16	555.1366 (100.0), 405.0842 (48.6), 387.0715 (38.7), 369.0602 (40.7), 339.0494 (34.6), 327.0508 (64.2), 303.0497 (86.4), 273.0395 (83.5), 163.0376 (36.2)
4	Dimethoxy-galloyl-mangiferin	C_28_H_26_O_15_	13.2	218, 258, 318, 373	603.1341	1.5	601.32	603.1328 (45.5), 405.0816 (100.0), 369.0609 (37.9), 327.0483 (49.5), 303.0494 (87.0), 285.0388 (34.6), 273.0398 (95.3), 245.1378 (41.2), 181.0486 (44.2), 139.0384 (51.5)
5	*p*-Hydroxybenzoylmangiferin derivative	C_26_H_22_O_13_	14.8	219, 258, 318, 373	543.1132	1.1	541.27	543.1133 (64.3), 423.0711 (13.7), 327.0498 (13.0), 303.0499 (38.6), 285.0395 (100.0), 273.0393 (23.8), 257.0445 (30.2), 121.0279 (56.9)
6	Vanilloylmangiferin derivative	C_27_H_24_O_14_	15.0	218, 258, 318, 373	573.1235	1.6	571.20	573.1229 (14.6), 313.0912 (5.0), 303.0494 (7.7), 285.0394 (35.6), 273.0389 (6.1), 257.0446 (8.2), 151.0386 (100.0)
7	2′-*O-trans*-caffeoylmangiferin	C_28_H_24_O_14_	15.1	219, 258, 318, 373	585.1238	1.0	583.28	585.1240 (26.9), 423.0923 (22.9), 327.0499 (11.7), 303.0500 (25.9), 285.0395 (28.0), 273.0395 (20.7), 257.0444 (9.4), 163.0388 (100.0)
8	*p*-Hydroxybenzoylmangiferin derivative	C_26_H_22_O_13_	15.2	219, 258, 318, 373	543.1131	1.3	541.53	543.1128 (78.9), 369.0603 (24.4), 339.0498 (69.1), 327.0496 (21.3), 303.0498 (23.2), 285.0391 (53.5), 274.0430 (18.7), 273.0392 (100.0), 121.0278 (65.3)
9	Caffeoylmangiferin derivative	C_28_H_24_O_14_	15.4	219, 258, 318, 373	585.1238	1.0	583.28	585.1236 (1.1), 387.0707 (12.5), 369.0604 (14.4), 357.0602 (13.7), 339.0497 (22.9), 327.0500 (11.2), 303.0498 (18.5), 273.0393 (55.9), 163.0389 (100.0)
10	Mangiferin derivative	C_25_H_22_NO_12_	15.5	208, 218, 258, 315, 373	528.1133	1.7	527.62	528.1130 (72.3), 510.1011 (52.8), 369.0603 (74.7), 351.0476 (41.4), 327.0487 (46.0), 303.0503 (100.0), 299.0548 (43.3), 273.0392 (61.6), 124.0387 (89.8)
11	2′-*O-trans*-coumaroylmangiferin	C_28_H_24_O_13_	15.8	208, 226, 258, 314, 373	569.1292	0.5	567.47	569.1292 (26.3), 327.0499 (5.7), 303.0501 (15.6), 285.0395 (24.3), 273.0395 (9.8), 257.0446 (8.2), 147.0437 (100.0)
12	Mangiferin derivative	C_30_H_28_O_15_	15.9	222, 258, 316, 369	629.1497	1.4	627.34	629.1501 (9.4), 303.0493 (8.4), 285.0392 (9.1), 273.0397 (4.8), 207.0653 (100.0), 175.0390 (36.2), 147.0437 (19.2), 119.0482 (5.1)
13	Metoxi*-O-*caffeoylmangiferin derivative	C_29_H_26_O_14_	16.0	220, 258, 316, 369	599.1396	0.7	597.53	599.1394 (13.1), 303.0499 (8.9), 285.0395 (11.4), 273.0396 (4.7), 257.0448 (3.1), 177.0547 (100.0), 149.0595 (4.1), 145.0281 (31.5)
14	Coumaroylmangiferin derivative	C_28_H_24_O_13_	16.3	221, 258, 317, 369	569.1289	1.0	567.15	569.1289 (32.8), 387.0712 (9.3), 369.0605 (12.8), 339.0499 (23.9), 303.0500 (11.3), 273.0394 (39.7), 163.0388 (9.8), 147.0438 (100.0)
15	Coumaroylmangiferin derivative	C_28_H_24_O_13_	16.4	221, 258, 316, 369	569.1290	0.9	567.54	569.1289 (35.0), 387.0709 (10.0), 369.0604 (12.8), 339.0499 (24.1), 303.0500 (11.3), 289.0342 (8,5), 273.0394 (40.6), 147.0438 (100.0)
16	Caffeoylmangiferin derivative	C_28_H_24_O_14_	16.6	221, 258, 316, 369	585.1239	0.8	583.41	585.1240 (13.7), 405.0829 (10.5), 369.0604 (18.5), 357.0602 (13.7), 303.0498 (63.0), 285.0400 (13.0), 273.0396 (18.6), 163.0389 (100.0), 145.0280 (11.6)
17	Benzoylmangiferin derivative	C_26_H_22_O_12_	16.9	221, 258, 318, 369	527.1180	1.7	525.46	527.1180 (41.3), 407.0765 (18.9), 327.0502 (6.9), 285.0394 (100.0), 273.0391 (11.2), 267.0862 (14.1), 261.0396 (6.1), 257.0440 (30.1)
18	Caffeoylmangiferin derivative	C_28_H_24_O_14_	17.1	221, 258, 318, 369	585.1239	0.8	583.18	585.1235 (24.2), 405.0816 (13.7), 369.0607 (28.6), 351.0496 (10.4), 327.0499 (15.0), 303.0501 (81.3), 285.0400 (11.2), 273.0395 (23.9), 163.0389 (100.0)
19	*p*-Hydroxybenzoylmangiferin derivative	C_26_H_22_O_13_	17.4	221, 258, 318, 369	543.1131	1.3	541.34	543.1108 (31.7), 423.0730 (9.7), 303.0497 (100.0), 285.0398 (52.7), 273.0396 (24.3), 257.0445 (20.5), 121.0279 (30.7)
20	Cinnamoylmangiferin derivative	C_28_H_24_O_12_	17.5	221, 258, 318, 369	553.1339	1.3	551.53	553.1350 (19.4), 303.0497 (16.3), 285.0396 (36.9), 273.0394 (11.1), 257.0456 (9.8), 163.0389 (16.4), 131.0488 (100.0)
21	Mangiferin derivative	C_33_H_36_O_15_	17.7	221, 258, 318, 369	673.2122	1.5	671.36	673.2146, 423.0917 (77.8), 303.0488 (53.6), 285.0387 (52.6), 233.1165 (70.1), 215.1064 (70.0), 205.1228 (66.4), 187.1119 (96.8), 173.0957 (100.0), 145.1011(98.6) 143.0701 (56.2)
22	Coumaroylmangiferin derivative	C_28_H_24_O_13_	17.7	221, 258, 316, 369	569.1290	0.9	567.47	569.1303 (19.2), 357.0597 (25.6), 303.0491 (52.6), 274.0411 (17.4), 273.0390 (78.6), 163.0377 (25.6), 147.0440 (100.0)
23	*p*-Hydroxybenzoylmangiferin derivative	C_26_H_22_O_13_	17.8	221, 258, 318, 369	543.1128	1.8	541.47	543.1129 (57.0), 423.0704 (14.0), 327.0489 (22.9), 303.0493 (48.6), 285.0400 (100.0), 283.0810 (22.8), 257.0444 (31.1), 121.0280 (92.1)
24	Caffeoylmangiferin derivative	C_28_H_24_O_14_	17.9	221, 258, 318, 369	585.1238	1.0	583.61	585.1245 (19.7), 423.0925 (9.6), 303.0498 (13.8), 285.0390 (27.4), 273.0394 (13.1), 257.0447 (8.4), 163.0389 (100.0), 145.0280 (11.2), 135.0435 (9.9)
25	Coumaroylmangiferin derivative	C_28_H_24_O_13_	18.2	221, 258, 316, 369	569.1289	1.0	567.41	569.1284 (20.3), 369.0601 (21.1), 327.0498 (9.9), 303.0500 (53.9), 273.0390 (14.1), 163.0384 (10.2), 147.0438 (100.0), 119.0487 (10.3)
26	2′-*O-trans*-cinnamoylmangiferin	C_28_H_24_O_12_	18.6	221, 258, 318, 369	553.1339	1.3	551.40	553.1338 (19.4), 433.0918 (10.3), 303.0497 (14.2), 285.0394 (63.5), 273.0395 (12.3), 257.0446 (16.5), 131.0488 (100.0)

**Figure 1:**
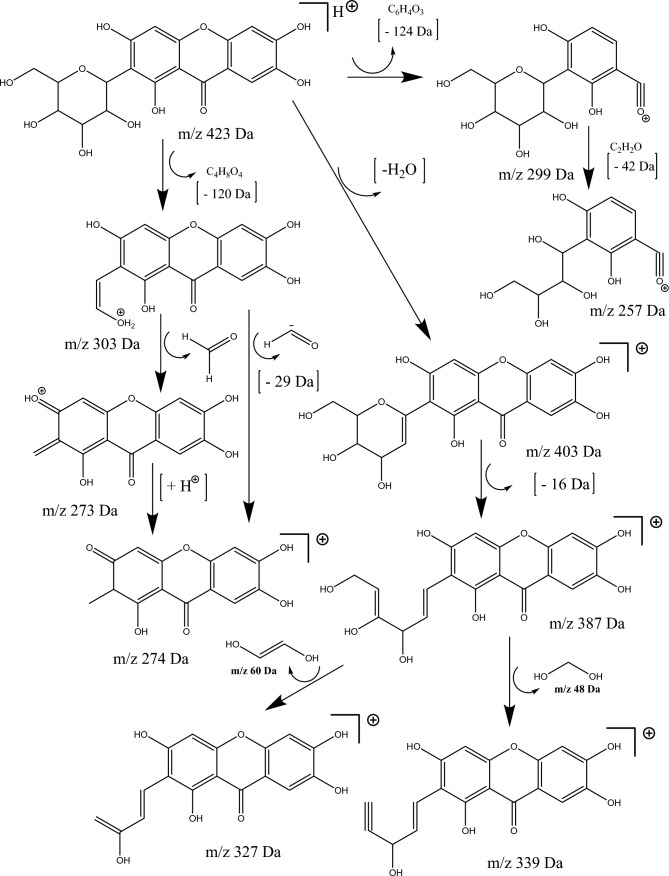
Fragmentation pathways of the mangiferin.

**Figure 2:**
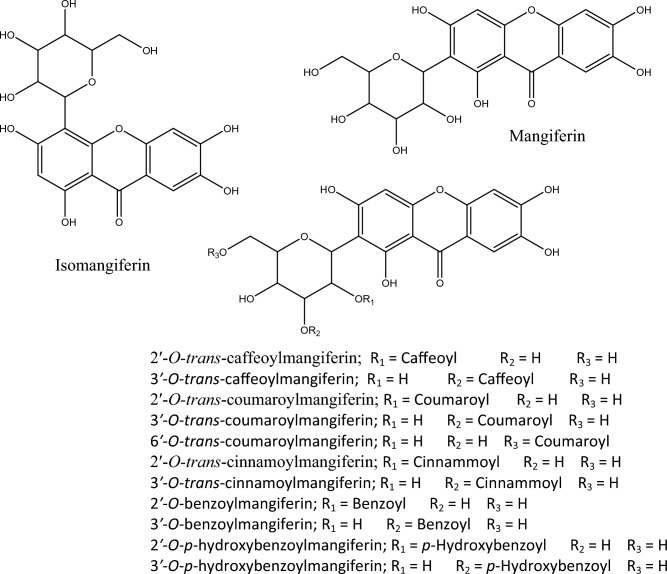
Xanthones present in extracts of *Fridericia* species.

In addition to the isomers **isomangiferin** (**1**) and **mangiferin** (**2**), four other groups of isomers with a molar mass of 542, 552, 568, and 584 Da have been characterized. All identified compounds are derivatives of mangiferin and/or isomangiferin, featuring at least two fragments present among the ions obtained from these two isomers in the second-order MS^2^ fragmentation analyses. A pathway leading to the detected fragments in the LC–MS analyses of mangiferin (compound **2**) has been proposed and is depicted in Fig. 1. Mangiferin and xanthones derivatives have been described as constituents of extracts from species of the Bignoniaceae family (Fig. 2)^6–9^.

Compounds **5**, **8**, **19**, and **23** exhibited protonated species with *m/z* 543 Da in the first-order MS1 analyses (Table 1). The fragments obtained in the MS^2^ analyses suggest that these compounds have a structure similar to ***p*-hydroxybenzoylmangiferin**. However, these isomers have different retention times and some distinct fragments among themselves. Additionally, compounds 5 and 23 showed a base peak at m/z 285 Da, while compounds **8** and **19** displayed base peaks at *m/z* 273 and 303 Da, respectively (Table 1). Pauletti et al.^6^ isolated the isomer **2'*-O-p-*hydroxybenzoylmangiferin** from the stem extract of *F. samydoides*, while Martin et al.^7^ isolated the isomer **3'*-O-p-*hydroxybenzoylmangiferin** from the leaves extract of *F. patellifera*.

Compounds **20** and **26** exhibited protonated species with an m/z of 553 Da and a base peak at *m/z* 131 Da in the MS^1^ analyses (Table 1). Fragmentation analyses and comparison with data from an authentic sample of **2'-*O-trans-*cinnamoylmangiferin** allowed for the identification of compound 26 as the derivative **2'-*O-trans*-cinnamoylmangiferin**, while compound 20 was determined to be an isomer with a similar structure. The compound **2'*-O-trans-*cinnamoylmangiferin** has previously been isolated from the fruits of *F. formosa* and the stems of *F. samydoides*. Martin et al.^7^ isolated the isomer **3′*-O-trans-*cinnamoylmangiferin** from the leaves extract of *F. patellifera*.

In the data analysis of compounds **11**, **14**, **15**, **22**, and **25**, protonated species with an *m/z* of 569 Da were observed (Table 1). These compounds exhibited a base peak at *m/z* 147 Da, characteristic of the loss of a coumaric acid residue. Compound **11** was identified as **2'*-O-trans*-coumaroylmangiferin** through comparison with data from an authentic sample. Fragmentation analyses of the other four isomers suggest that they are substances with similar structures (Table 1). **2'-*O-trans-*coumaroylmangiferin** has previously been isolated from the leaves extract of this species by Brandão et al.^8^. Pauletti et al.^6^ also isolated this compound from the stem extract of *F. samydoides*. Furthermore, Martin et al.^7^ isolated the isomers **3'*-O-trans-*coumaroylmangiferin** and **6'*-O-trans-*coumaroylmangiferin** from the leaves of *F. patellifera*.

Compounds **7**, **9**, **16**, **18**, and **24** exhibited a base peak at *m/z* 163 Da, indicating the presence of an additional caffeic acid residue in the structure of mangiferin. These isomers showed protonated species with an *m/z* of 585 Da. Fragmentation analyses revealed that, despite sharing similar mass-to-charge ratio fragments, there are differences in the molecule's fragmentation pattern as these isomers do not entirely share the same ions formed. Compound **7** was identified as **2'*-O-trans-*caffeoylmangiferin** through comparison with an authentic sample. This substance has been previously isolated from this species and also from *F. samydoides*^6,8^. The isomer **3'*-O-trans-*caffeoylmangiferin** was isolated from the leaves extract of *F. patellifera*^7^.

The exact mass obtained for compound **3** was 555.1339 Da, suggesting a molecular formula of C_24_H_26_O_15_, which is structurally compatible with mangiferin with the addition of a pentose residue (such as arabinose, apiose, or xylose, for example). Fragmentation analyses revealed the presence of fragments with *m/z* values of 405, 387, 339, 327, 303, and 273 Da, indicating that part of the molecular structure is similar to mangiferin (Table 1). The data obtained in this study, along with a comparison with literature data on xanthones, suggest that compound 3 is possibly a new derivative of mangiferin^18,19^.

Compounds **4** and **6** exhibited protonated species with *m/z* values of 603 and 573 Da, respectively. The base peaks were at m/z 405 Da for compound 4 and *m/z* 285 Da for compound 6 (Table 1). These compounds share similar fragments when the mangiferin structure is fragmented (Fig. 1). Additionally, in the fragmentation of compound **4**, a fragment with *m/z* 181 Da related to the loss of a syringic acid residue is obtained, while in compound 6, a fragment with *m/z* 151 related to the loss of a vanillic acid residue is obtained. Data analysis suggests that these compounds are two derivatives of mangiferin linked to syringic and vanillic acid residues. A search of literature databases did not identify any records of mangiferin derivatives with these structures, suggesting that these are novel substances."

At a retention time of 16.0 min, a compound (13) with an *m/z* of 599 Da is observed. In the MS^2^ analyses, a base peak of 177 Da and a fragment of 149 Da, attributed to the loss of a methoxylated caffeic acid residue (ferulic or isoferulic acid), are obtained. Additionally, fragments related to the mangiferin structure residue were observed (m/z values of 303, 273, 257 Da) (Table 1). Literature data records a mangiferin derivative with a similar structure synthesized from mangiferin^20^. Therefore, compound **13** has been partially characterized as **methoxy*-O-*caffeoylmangiferin**, marking the first identification of this substance in a natural source.

Compound **17**, with a retention time of 16.9 min, exhibited a protonated species with an *m/z* of 527 Da. In the MS^2^ analyses, a base peak at *m/z* 285 Da and fragments similar to those found in mangiferin derivatives were observed. Martin et al.^7^ isolated **3'*-O-*benzoylmangiferin** from the leaf extract of *F. patellifera*. Therefore, compound **17** has been partially characterized as Benzoylmangiferin.

Finally, three compounds (**10**, **12**, and **21**) eluted at retention times of 15.5, 15.9, and 17.7 min with protonated species of *m/z* 528, 629, and 673 Da, respectively. These substances exhibited a series of fragments similar to those observed for mangiferin and its derivatives, suggesting that these compounds also share some structural features (Table 1 and Fig. 1). Therefore, it was concluded that these are three additional derivatives of the xanthone mangiferin.

### Quantification of the major xanthones

**Mangiferin**, **2′-*O-trans*-caffeoylmangiferin** and **2′-*O-trans*-coumaroylmangiferin** have already been identified and isolated from leaves, stems, and fruits of *F. formosa*^8^. However, these xanthones had not yet been quantified in this specie. The method developed to quantification of xhantones in *F. formosa* extract was precise and accurate since it showed an RSD value minor of 5%. The method was linear in the range of 30 to 300 µg/mL with limits of detection and quantification of 3.67 and 11.11 µg/mL, respectively (Table 2).

**Table 2:** Precision, accuracy and linearity parameters of xanthones quantification method.

Precision (repeatability)		
Concentration (µg/mL)	RSD	
30	3.98	
50	1.46	
100	1.23	
150	1.63	
300	0.27	

Accuracy		
Concentration added (µg/mL)	Recovery (%)	RSD
50	100.87	2.46
100	98.09	3.22
150	101.04	2.90

Linearity	Mangiferin in methanol	Mangiferin in matrix
r^2^	0.9999	0.9994
Equation	y = 39437x − 157,466	y = 40538x − 126,934
Significance (a ≠ 0)	*p* = 0.00002 (a ≠ 0)	*p* = 0.00003 (a ≠ 0)
Linearity desviation	*p* = 1.00000 (linear)	*p* = 0.90000 (linear)

*r*^*2*^ R-Squared (coefficient of determination), *p* P-value (level of statistical significance), *RSD* relative standard deviation.

Moreover, the other constituents of the extract do not affect the analytical response since the standard curves obtained in methanol and in the fortified extract (matrix) were parallel and percentage calculations of the matrix effect (%ME) resulted in 2.72%, considered as a very low matrix effect (%ME < 20%)^21,22^.

Therefore, the validated method was used to quantify the three major xhantones in extract of *F. formosa* leaves. The xanthones content were 96.5 ± 0.6 mg/g (9.65% w/w) for **mangiferin**, 106.8 ± 0.2 mg/g (10.68% w/w) for **2′-*O*-trans-caffeoylmangiferin** and 34.1 ± 0.1 mg/g (3.41% w/w) for **2′-*O-trans*-coumaroylmangiferin**.

The mangiferin quantity is greater than that found in methanol extract of *Mangifera foetida* leaves (0.28% w/w), species whose antiviral activity has already been reported^23,24^. 2′-*O-trans*-caffeoylmangiferin and 2′-*O-trans*-coumaroylmangiferin had not yet been quantified in other plant species.

### Cytotoxic effect and antiviral activity

The ethanolic extract and isolated compounds from the species *F. formosa* were evaluated for cytotoxicity in Vero cells, with cytotoxic concentration values (CC_50_) ranging between 400 and 99.5 µg/mL.

Antiviral activity tests were conducted against Chikungunya, Mayaro, and Zika viruses at non-cytotoxic concentrations for the ethanolic extract and isolated compounds at concentrations ranging from 400 to 0.781 µg/mL (Table 3). Only anti-MAYV activity was observed for the ethanolic extract, with an effective antiviral concentration (EC_50_) of 36.1 µg/mL. The compounds **2′-*O*-*trans*-coumaroylmangiferin** and **2′-*O*-*trans*-cinamoylmangiferin** also inhibited the Mayaro virus multiplication cycle, with EC_50_of 180.6 µg/mL and 149.4 µg/mL, respectively. Literature data show the antiviral potential of xanthones obtained from different natural sources, including xanthones obtained from the species *F. formosa*. These studies have reported that xanthones with different chemical structures inhibit the multiplication cycle of Vaccinia virus (VACV-WR), dengue virus (DENV-2), hepatitis C virus (HCV), influenza virus (H1N1), herpes simplex virus types 1 (HSV-1), and 2 (HSV-2) viruses^8,25,26^. However, this report presents the first evidence of the antiviral activity of *F. formosa* extract and its xanthones against Mayaro virus.

**Table 3:** Antiviral activity of extract and isolated xanthones from *Fridericia formosa* leaves.

	Cytotoxic (CC_50_)	Antiviral activity (EC_50_)			
	VERO µg/mL	ZIKV µg/mL	CHIKV µg/mL	MAYV µg/mL	SI
Ethanol extract	170.0 ± 1.3	NA	NA	36.1 ± 1.7	4.7
Mangiferin	172.1 ± 1.6	NA	NA	NA	
2′-*O-trans*-caffeoylmangiferin	> 400	NA	NA	NA	
2′-*O-trans*-coumaroylmangiferin	> 400	NA	NA	180.6 ± 1.8	> 2.2
2′-*O-trans*-cinnamoylmangiferin	99.5 ± 1.6	NA	NA	149.4 ± 2.7	0.7

*NA* not active, *CC*_*50*_ 50% cytotoxic concentration, *EC*_*50*_ 50% effective concentration, *SI* selectivity index.

The EC_50_ observed for Mayaro virus were higher than those reported for dengue virus: 40.4 µg/mL and 3.5 µg/mL for **2′-*O*-*trans*-coumaroylmangiferin** and **2′-*O*-*trans*-cinamoylmangiferin**, respectively^8^. This difference among results can be attributed to the fact that Mayaro and Dengue are different arboviruses.

The selectivity index (SI) is a parameter that can predict compounds with promising biological activity because this parameter infers on the potency and possible selectivity for the development of new drugs^27^. Furthermore, according to Koch et al.^28^, SI values below 2.0 may indicate that the tested samples, although exhibiting biological activity, could have toxic properties that compromise their benefits. Therefore, the compound **2*′-O-trans-*coumaroilmangiferin** might be more promising as it has an SI greater than 2.2. The extract also showed high selectivity (SI 4.7) and its other components, which were not isolated, could also contribute to the observed antiviral activity, since the extract showed an EC_50_ lower than the isolated xanthones.

## Materials and methods

### General procedures

In liquid chromatography analyses, analytical-grade solvents from Merck (methanol, acetonitrile, and formic acid (Merck®, Kenilworth, NJ, USA)) were used. Reference samples of mangiferin, 2'*-O-trans-*cinnamoylmangiferin, 2'*-O-trans-*cummaroylmangiferin, and 2'*-O-trans-*caffeoylmangiferin were obtained from the collection of the Medicinal Chemistry Laboratory at the School of Pharmacy, Federal University of Ouro Preto, with spectrometric data published by Brandão et al.^8^. Plastic consumables used in the biological assays (centrifuge tubes, pipette tips, plates, and cell culture bottles) were all from the Kasvi**®** brand. Dulbecco's Modified Eagle's Medium–high glucose, trypsin, fetal bovine serum, penicillin/streptomycin, and amphotericin B were sourced from Sigma-Aldrich (Sigma-Aldrich®, San Luis, Missouri, EUA).

### Plant material and chemicals

The leaves of *F. formosa* were collected in Belo Horizonte, state of Minas Gerais, Brazil. The species was identified by Dr. J. A. Lombardi, Department of Botany, Institute of Biosciences, UNESP, Rio Claro, Brazil. A voucher specimen was deposited in the BHCB/UFMG, Belo Horizonte, Minas Gerais, Brazil, under the number 23885.

### Extract preparation

The ethanolic extract was obtained as described by Brandão et al.^3^ with minor modifications. The leaves (241.0 g) were dried in a ventilated oven at 40 °C for 72 h, ground and exhaustively extracted by percolation with ethanol at room temperature for 48 h each time. A crude dark-green ethanol extract residue (EEFFL, 26.45 g) was obtained in a rotatory evaporator under reduced pressure at 50 °C. The ethanolic extract was subjected to antiviral activity tests.

### Liquid chromatography–mass spectrometry in series (LC-DAD-ESI–MS)

Ethanolic extract from *F. formosa* leaves was dissolved in methanol and filtered through a 0.22 µm microfilter. The UPLC ACQUITY system (Waters) was used, coupled with a DAD detector—scanning from 220 to 400 nm—and a mass spectrometry detector (MS), with an HSS C18 column (1.7 μm particles, 50 × 3 mm i.d.), operating at a flow rate of 0.3 mL/min and maintaining the column oven at 40 °C. A gradient of H_2_O (0.1% formic acid) and ACN (0.1% formic acid) was applied, involving a long linear elution period (5–95% H_2_O over 10 min), followed by a short isocratic elution period (95% ACN for 1 min). In obtaining the mass spectra, an Electrospray ionization (ESI) system was used, with a capillary temperature of 250 °C, the spray voltage set at 3.5 kV, capillary voltage at + 3 V and − 47 V for positive and negative polarities respectively, and a tube lens offset of 0 V and − 25 V for positive and negative polarities respectively. Nitrogen was used as the collision gas with a flow rate of 50 arbitrary units. Mass analysis was conducted in full scan mode from 100 to 1500 Da in negative mode.

### High-resolution mass spectrometry analyses

The UHPLC-HRMS/MS chromatographic analyzes were conducted as previously described with minor modifications (Cruz et al.^13^). A Nexera UHPLC system (Shimadzu) coupled to a high-resolution mass spectrometer (maXis ETD high-resolution ESI-QTOF, Bruker) controlled by Compass 1.7 software (Bruker). A Shimadzu Shim-Pack XR-ODS-III column (C18, 2.2 μm, 2.0 × 150 mm) was used with a column oven temperature set at 40 °C and a mobile phase flow rate of 400 μL/min. Mobile phases A and B (MilliQ water and acetonitrile, respectively) added 0.1% formic acid to each of the mobile phases. Elution programming began with an initial gradient of 5% B over 5 min, followed by a linear gradient to 100% acetonitrile over 40 min. Immediately after reaching the 100% gradient, the same conditions were maintained for another 5 min. Detection was performed UV-PDA (190–450 nm), mass spectra were acquired in positive mode at a rate of 5 Hz. Detection of the compound was obtained by chromatographic dissection of the peak with subsequent determination of the molecular formula according to the exact mass and isotopic standard (MS1). Putative identification was based on comparison of compound fragment spectra (MS2) with reference spectra from an internal standard compound database (FIOCRUZ-Minas), the MassBank public spectrum database, and in-silico fragment spectra. generated from the Universal Natural Products Database^6,29^.

### Quantification of the major xanthones

The analysis for quantification of xanthones was performed on a Waters Alliance HPLC–UV system (Waters) equipped with a C-18 column (LiChrospher, 4.0 × 125 mm, 5 μm particle size) (Merk) at 40 °C. The mobile phase comprised 0.1% formic acid in Milli-Q purified water (solvent A) and acetonitrile (solvent B). The flow rate was 1.0 mL/min and the injected volume was 10 µL, using the gradient (10–18% B/ 0–5 min, 18–21% B/ 5–12 min, 21–100% B/ 12–15 min, 100–10% B/ 15–18 min, 10% B/ 18–20 min). 254 nm wavelength was used for the analysis.

The extract solutions were prepared in methanol at the concentration of 1 mg/mL. Mangiferin was used as standard at a range of concentration of 30–300 μg/mL.

The analytical method was validated to demonstrate the matrix effect, linearity, precision (repeatability), accuracy and limits of detection and quantification^30^.

### Cell culture and virus

Vero cells (kidney cells from *Cercopthecus aeothiops*, ATCC CCL-81 TM, Manassas, VA, USA) were cultured in Dulbecco’s modified Eagle’s medium (DMEM) at 37 °C, in a 5% CO_2_ atmosphere, supplemented with 5% fetal bovine serum (FBS), 100 U/mL penicillin/streptomycin and 5 µg/mL amphotericin B. Zika, Chikungunya and Mayaro viruses were kindly donated by Dra. E. Kroon (UFMG, Belo Horizonte, Brazil), Dr. E. Arruda Neto (UNIFESP, São Paulo, Brazil) and Dr. M. Nogueira (FARMEP, São Paulo, Brazil), respectively. The virus titers were 6.8 × 10^5^ PFU/mL, 1.44 × 10^11^ PFU/mL and 2.5 × 10^8^ PFU/mL for ZIKV, CHIKV and MAYV, respectively^3,31,32^.

### Cytotoxicity assay

The cell suspensions were distributed into 96-well microplates, with each well containing 6.0 × 10^4^ cells. The plates were incubated in a humidified atmosphere with 5% CO_2_ at 37 °C for 24 h. The samples solubilized in pure dimethyl sulfoxide (DMSO) were diluted in culture medium supplemented with 1% fetal bovine serum (FBS)^33,34^. The substance dilutions ranged from 400 to 0.781 µg/mL. After the formation of a cell monolayer at the bottom of the wells, the culture medium was removed, and 100 μL of the diluted sample solutions, along with 100 μL of culture medium enriched with 1% FBS, were added. The plates were then incubated under the same atmospheric conditions^33,34^. The sample volumes were calculated to ensure that the DMSO concentration per well did not exceed 0.2%^35^.

After 72 h from the addition of the samples, the culture medium was removed, and 28 μL of MTT solution (2.0 mg/mL in PBS, Merck®, Kenilworth, NJ, USA) was added to each well. The plates were incubated again for 120 min. At the end of this period, 132 μL of pure DMSO was added to all wells, and the plates were shaken on a plate shaker for 15 min to dissolve the formazan formed^33,34^. The quantification of formazan, obtained by the reduction of the tetrazolium salt in viable cells, was performed using the VICTOR™ X3 microplate reader (Perkin Elmer®) with WorkOut 2.5 software at 490 ɳm. Cell proliferation was compared with the control group.

Cellular toxicity was expressed in terms of the 50% cytotoxic concentration (CC_50_). The cytotoxic percentage was calculated as [(A − B)/A] × 100, where A and B represent the optical densities at 490ɳm (OD_490_) of the wells containing untreated cells (A) and treated cells (B), respectively.

### Antiviral assays

The experiment following the methodology described by Brandão et al.^3^. Sample stock solutions were solubilized in dimethyl sulfoxide (DMSO) and analyzed at non-cytotoxic concentrations. Vero cell monolayers (6 × 10^4^ cells per well) were cultured in 96-well plates. After 24 h of incubation at 37 °C in 5% CO_2_, the culture medium (DMEM 5% FBS) was removed and 100 μL of sample dilutions were added to each well along with 100 μL of viral suspension. After treatment the plates were then incubated in a humidified atmosphere with 5% CO_2_ at 37 °C for 72 h. Cell viability was assessed using the MTT colorimetric technique^33^. then the supernatant was removed and 28.0 μL of an MTT solution (2.0 mg/mL in PBS) was added to each well. The plates were incubated for 2 h at 37 °C, and after this incubation period, 132.0 μL of DMSO was added to each well, aiming to dissolve the formazan crystals. Plates were homogenized for 15 min (using a New Brunswick Scientific C24 shaker) and optical density was determined at 490 ɳm (OD_490_) using a microplate reader. In all experiments, cell controls, virus controls and cytotoxicity controls were used. The effective concentration that exhibits 50% antiviral effect, known as the effective concentration at 50% (EC_50_), is expressed as the concentration that protects 50% of the treated cells from destruction caused by the virus. The percentage of protection was calculated as [(A − B)/(C − B)] × 100, where A, B, and C represent the OD_490_ of the wells containing treated and infected cells (A), untreated and infected cells (B), and untreated and uninfected cells (C), respectively. To calculate the selectivity index (SI), the ratio of the 50% cytotoxic concentration (CC_50_) to the 50% effective concentration (EC_50_) value was used.

### Statistical analyses

The data from cytotoxicity and antiviral assays were evaluated based on their means and standard deviations with statistical significance set at less than 0.05. The 50% cytotoxic concentration and the 50% effective concentration were determined in comparison with the control and obtained through non-linear regression. These analyses were conducted using the statistical package GraphPad Prism 7.00.

### Statement on permissions and/or appropriate licenses for the collection of plant specimens or seeds

This research was conducted in compliance with all mandatory requirements of current legislation. The studied plant is a native species of the Brazilian flora, and access to Genetic Heritage was registered in the NATIONAL SYSTEM FOR MANAGEMENT OF GENETIC HERITAGE AND ASSOCIATED TRADITIONAL KNOWLEDGE (SisGen) under number **AF2D451**, in accordance with the provisions of Brazilian Law No. 13.123/2015 and its regulations. This plant species is not listed as endangered.

## Conclusions

*F. formosa* leaves are rich in xanthones, with 26.05 ± 0.92 mg/g of dry plant material. In this study, twenty-six compounds derived from mangiferin were attributed, fourteen of which are described in the literature, these constituents are present in the extract of *F. formosa* leaves. Therefore, it can be suggested that this plant species is a source of new mangiferin derivatives. The ethanolic extract of the leaves has also been shown to be a potential source of antiviral compounds. The inhibition of the multiplication cycle of the MAYV virus was observed in in vitro assays, and antiviral action against Herpes, Vaccinia and Dengue 2 viruses is also found in the literature. The xanthones have a wide range of biological activities and may contribute to the observed antiviral activity.

### Supplementary Information

Supplementary Information.

## Data Availability

They were included in the support material.
