# Supplementary material for: Identification, characterization and quantification of xanthones from Fridericia formosa leaves extract with antiviral activity
Source: Sci Rep. 2024 Jan 26;14:2258. doi: 10.1038/s41598-024-51881-3 (PMC10817953; doi:10.1038/s41598-024-51881-3)
Supplement: Supplementary file 1 — Supplementary Information. [file 41598_2024_51881_MOESM1_ESM.docx]

**Identification, characterization and quantification of xanthones from** *Fridericia formosa* **leaves extract with antiviral activity**

Luana Beatriz Araújo Vaz^1^, Tatiane Roquete Amparo^1^, Adriana Cotta Cardoso Reis^1^, Breno de Mello Silva^2^, Cíntia Lopes de Brito Magalhães^2^, Markus Kohlhoff ^3^, and Geraldo Célio Brandão^1,*^

^1^Programa de Pós-graduação em Ciências Farmacêuticas, Escola de Farmácia, Universidade Federal de Ouro Preto, Campus Morro do Cruzeiro, Ouro Preto Zip code 35.402-163, Minas Gerais, Brazil;

^2^Departamento de Ciências Biológicas, ICEB, Universidade Federal de Ouro Preto, Campus Morro do Cruzeiro, Ouro Preto Zip code 35.402-163, MG, Brazil;

^3^Laboratório de Química de Produtos Naturais Bioativos, Fundação Oswaldo Cruz. Instituto René Rachou, Belo Horizonte Zip code 30.190-009, Minas Gerais, Brazil;

*Correspondence: celiobrandao@ufop.edu.br; Tel.: +55-31-3559-1088; Fax: +55-31-3559-1069

**Abstract**

*Fridericia formosa* is a neotropical liana species found in the Cerrado biome in Brazil. It has been of great interest to the scientific community due to its potential as a source of new antivirals, including xanthones derived from mangiferin. In this context, the present study aimed to characterize and quantify the xanthones present in the ethanol extract of this species using high performance liquid chromatography. Additionally, the antiviral activity against Chikungunya, Zika, and Mayaro viruses was evaluated. The chromatographic analyses partially identified twenty-six xanthones, among which only fourteen had already been described in the literature. The xanthones mangiferin, 2′-*O-trans*-caffeoylmangiferin, and 2*′-O-trans*-coumaroylmangiferin, are present in higher quantities in the extract, at concentrations of 9.65%, 10.68%, and 3.41% w/w, respectively. In antiviral assays, the extract inhibited the multiplication cycle only for the Mayaro virus with a CE_50_ of 36.1 μg/mL. Among the isolated xanthones,2′-*O-trans*-coumaroylmangiferin and 2*′-O-trans*-cinnamoylmangiferin inhibited the viral cytopathic effect with CE_50_ values of 180.6 and 149.4 μg/mL, respectively. Therefore, the extract from *F. formosa* leaves, which has a high content of xanthones, has antiviral potential and can be a source of new mangiferin derivatives.

**Compound (1) RT 11.0 min. MM 422 Da** *Isomangiferin*


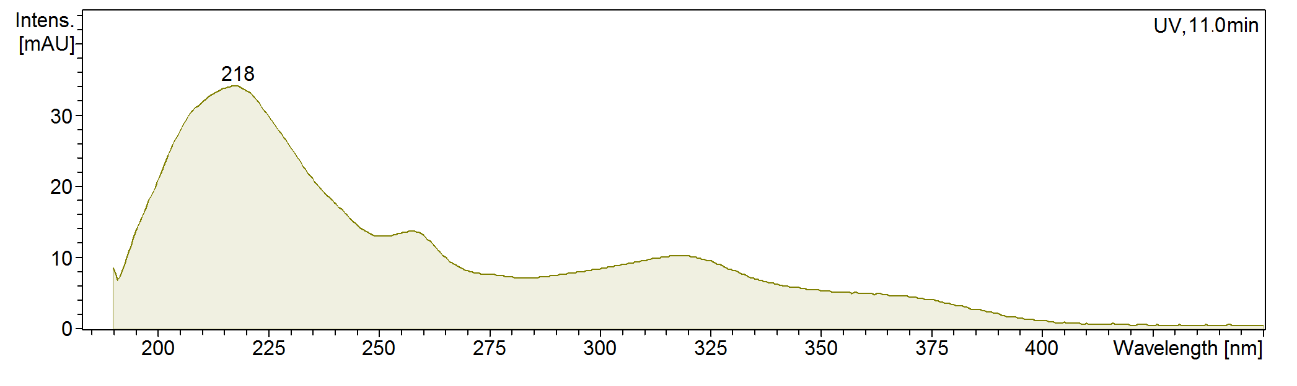


Figure 1S. UV spectrum of C-glycosyl xanthone present in the ethanolic extract of *F. formosa* leaves


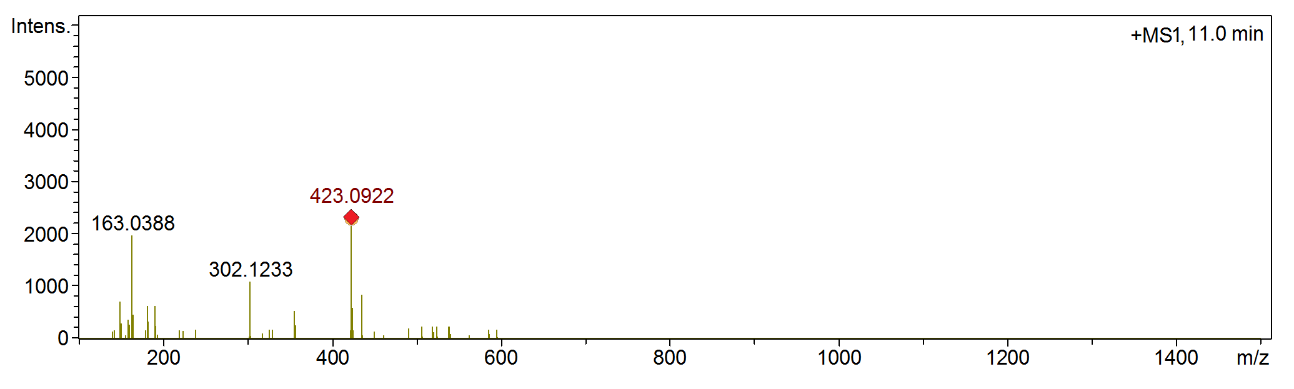


Figure 2S. MS^1^ spectrum of C-glycosyl xanthone present in the ethanolic extract of *F. formosa* leaves

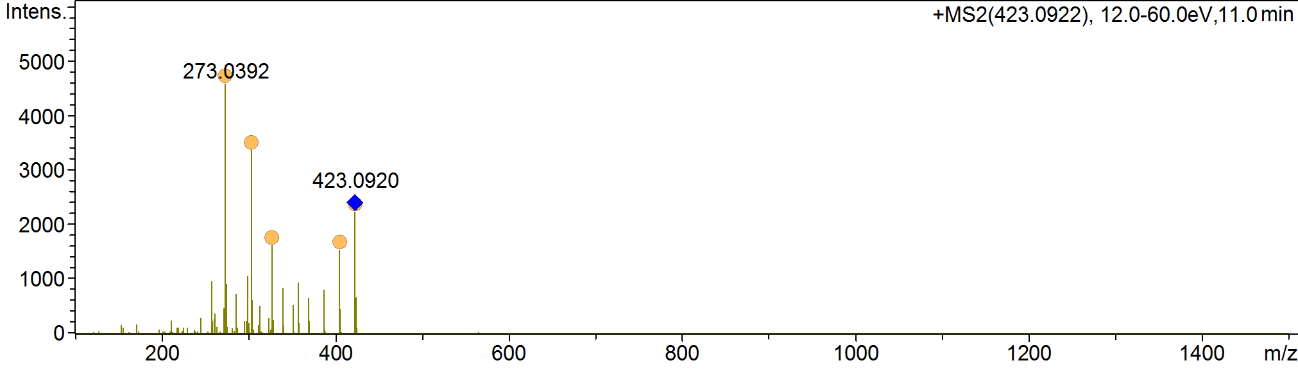


Figure 3S. MS^2^ spectrum of C-glycosyl xanthone present in the ethanolic extract of *F. formosa* leaves


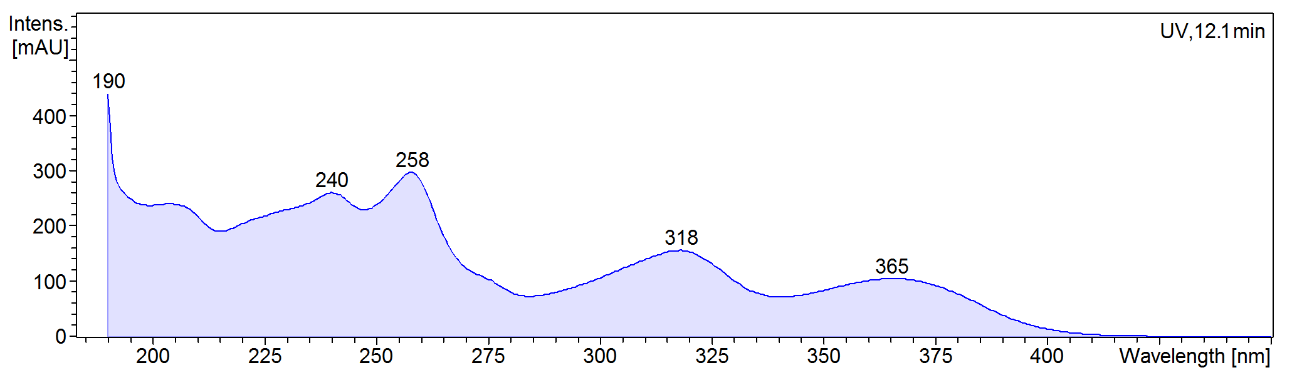
**Compound (2) RT 12.1 min. MM 422 Da** *Mangiferin*


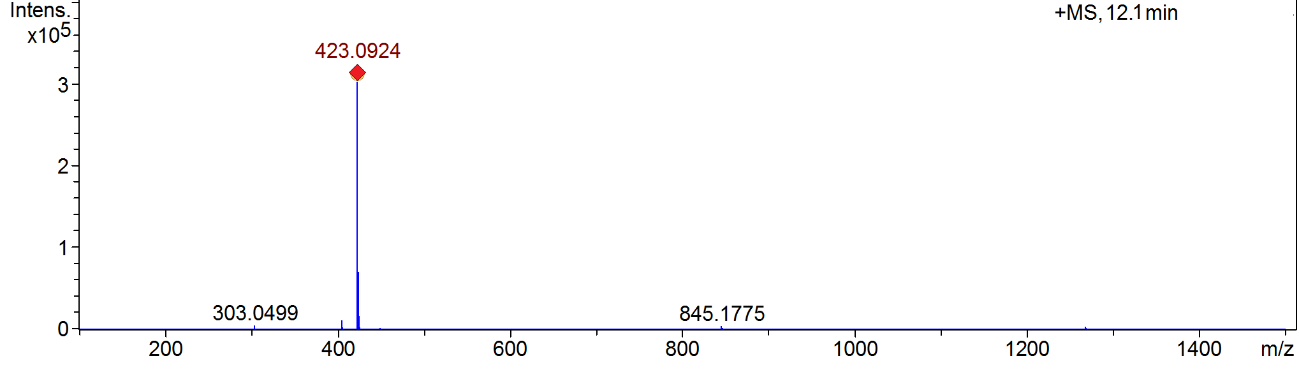


Figure 5S. MS^1^ spectrum of C-glycosyl xanthone present in the ethanolic extract of *F. formosa* leaves

Figure 4S. UV spectrum of C-glycosyl xanthone present in the ethanolic extract of *F. formosa* leaves


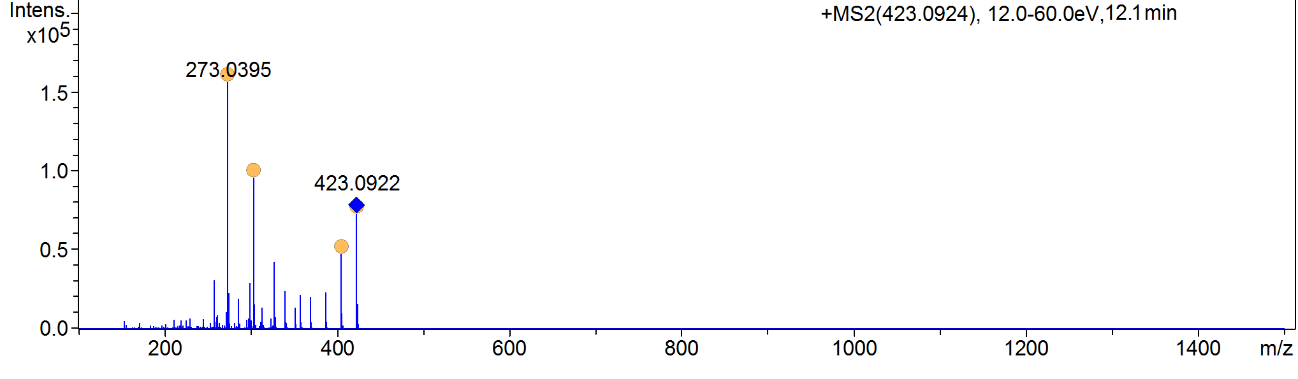


Figure 6S. MS^2^ spectrum of C-glycosyl xanthone present in the ethanolic extract of *F. formosa* leaves

**Compound (3) RT 12.6 min. MM 554 Da -** *Mangiferin-O-pentosyl derivative*

Figure 7S. UV spectrum of C-glycosyl xanthone present in the ethanolic extract of *F. formosa* leaves


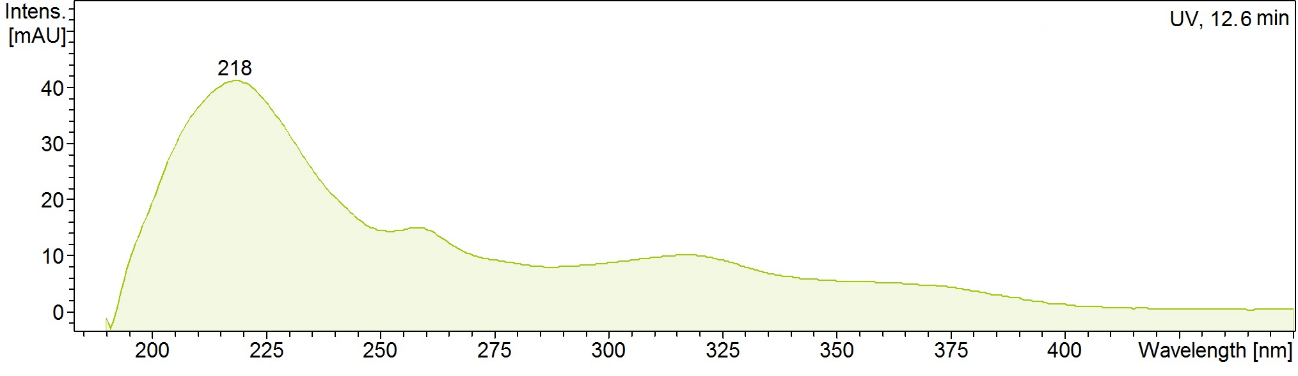


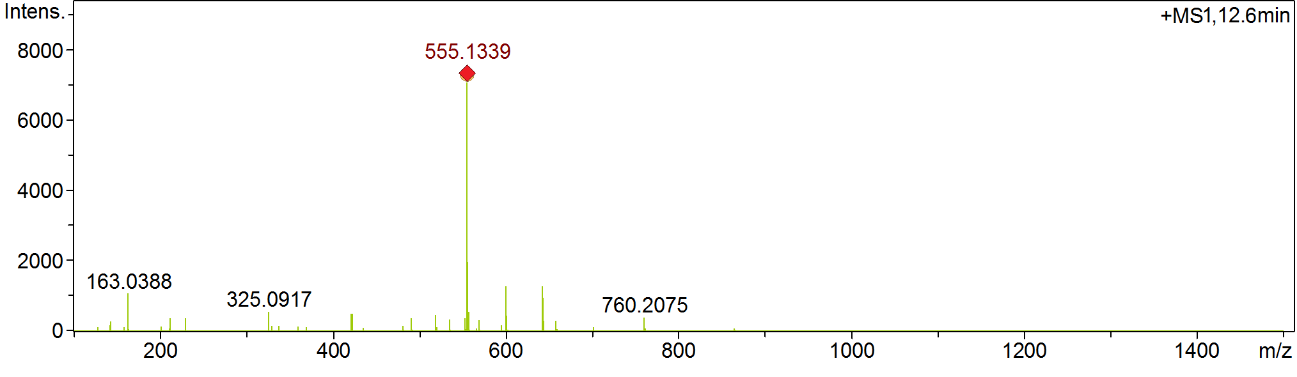


Figure 8S. MS^1^ spectrum of C-glycosyl xanthone present in the ethanolic extract of *F. formosa* leaves


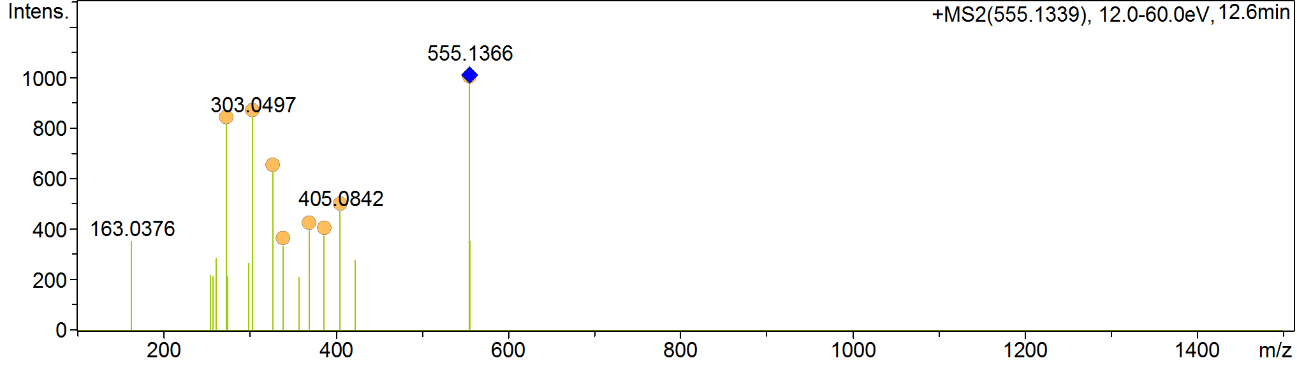


**+**

Figure 9S. MS^2^ spectrum of C-glycosyl xanthone present in the ethanolic extract of *F. formosa* leaves

**Compound (4) RT 13.2 min. MM 602 Da -** *Dimethoxy-galloyl-mangiferin*


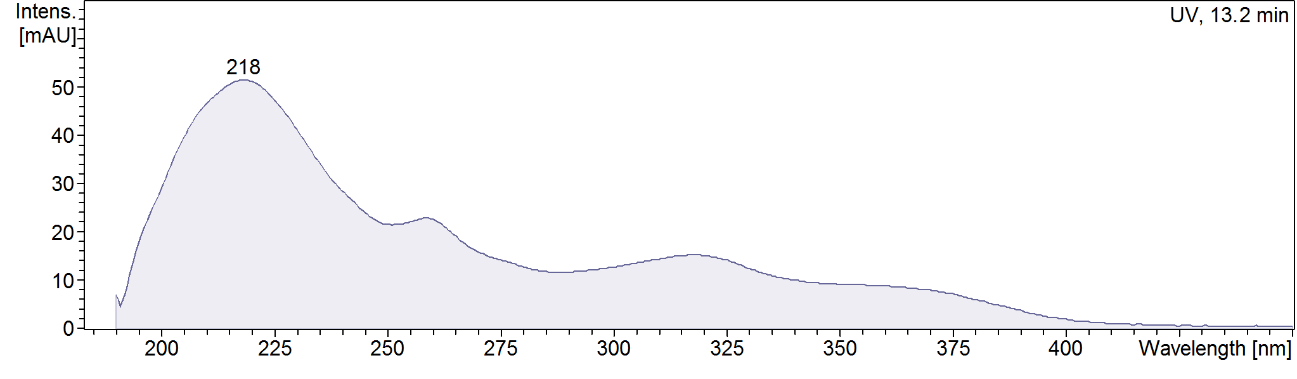


Figure 10S. UV spectrum of C-glycosyl xanthone present in the ethanolic extract of *F. formosa* leaves


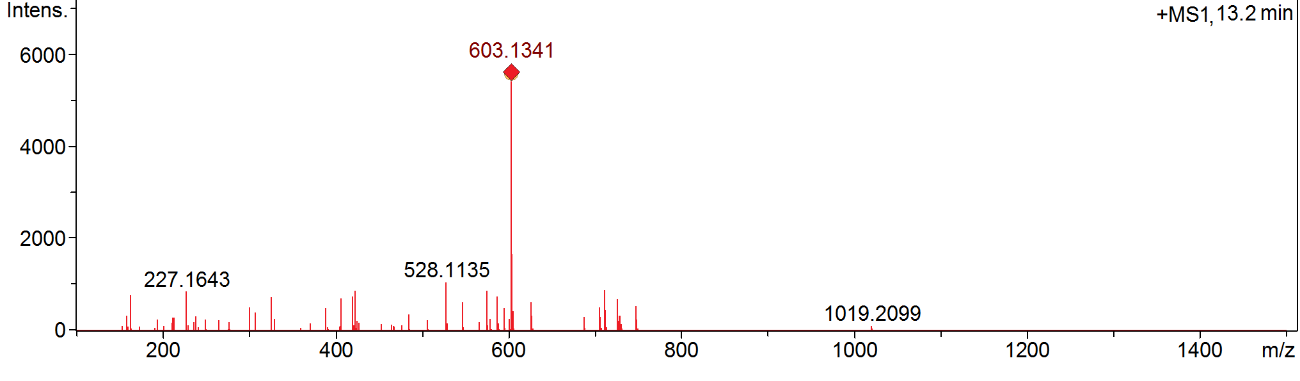


**+**

Figure 11S. MS^1^ spectrum of C-glycosyl xanthone present in the ethanolic extract of *F. formosa* leaves


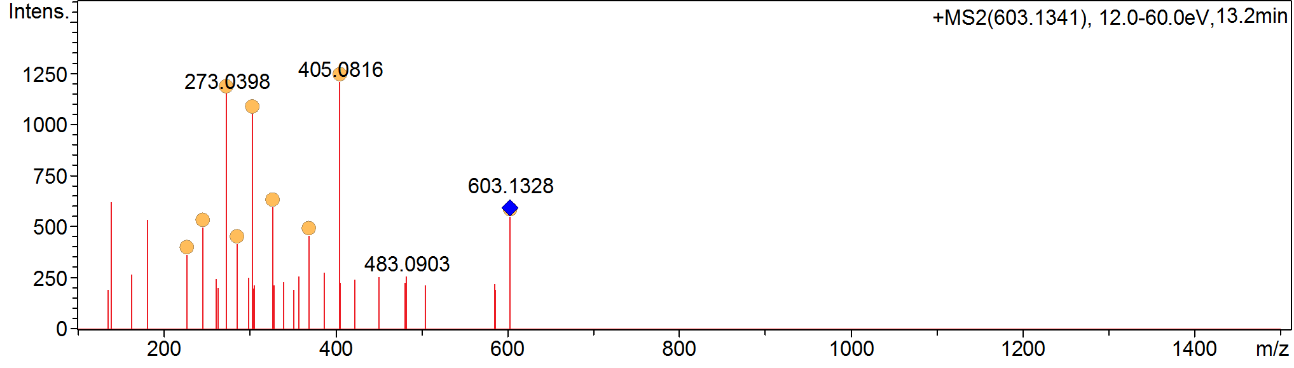


Figure 12S. MS^2^ spectrum of C-glycosyl xanthone present in the ethanolic extract of *F. formosa* leaves

**Compound (5) RT 14.8 min. MM 542 Da -** *p-Hydroxybenzoylmangiferin derivative*


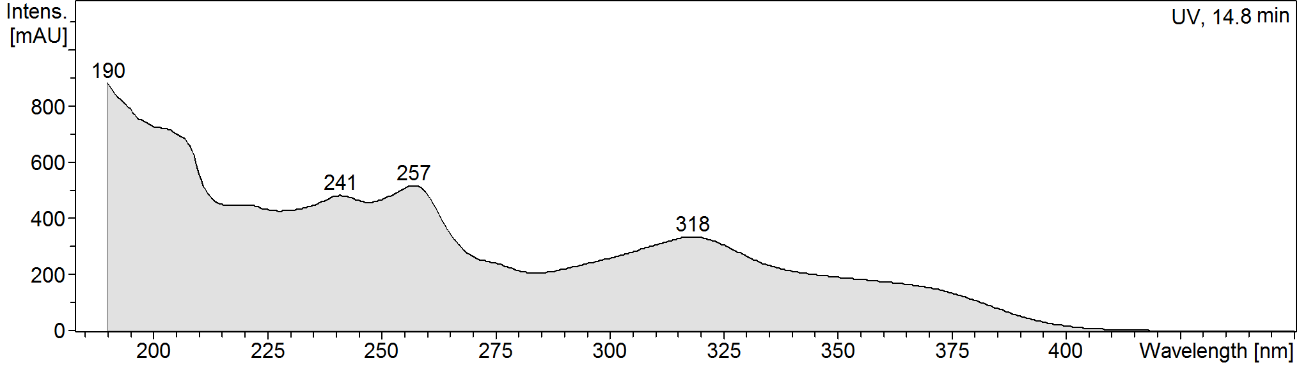


Figure 13S. UV spectrum of C-glycosyl xanthone present in the ethanolic extract of *F. formosa* leaves

Figure 14S. MS^1^ spectrum of C-glycosyl xanthone present in the ethanolic extract of *F. formosa* leaves


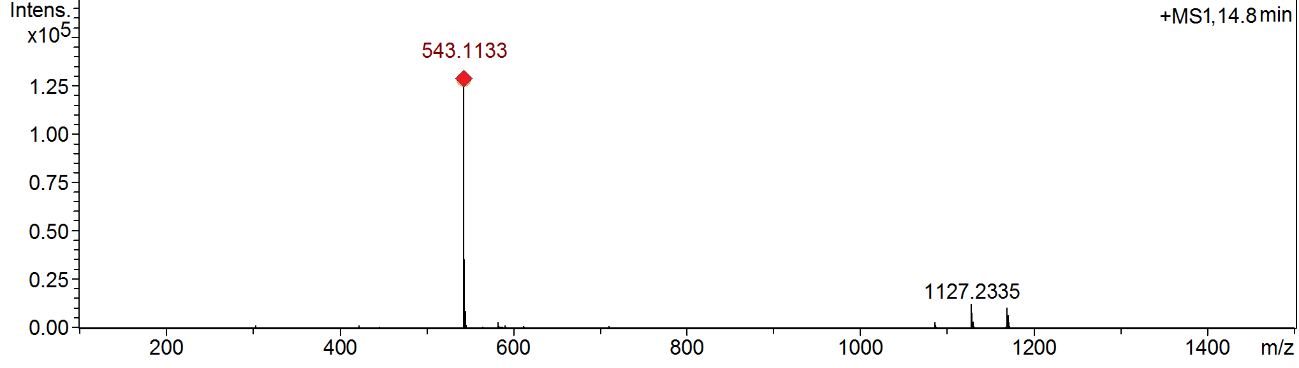


**+**


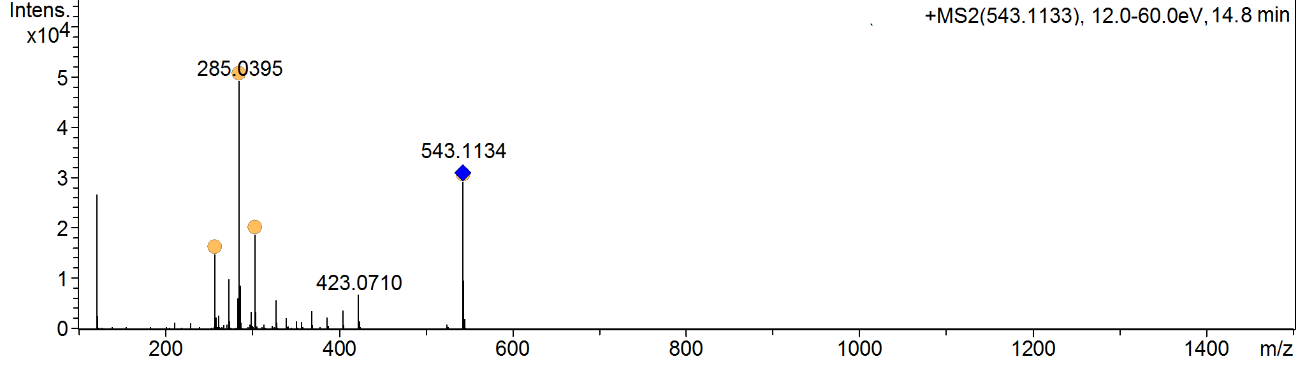


Figure 15S. MS^2^ spectrum of C-glycosyl xanthone present in the ethanolic extract of *F. formosa* leaves

**Compound (6) RT 15.0 min. MM 572 Da -** *Vanilloylmangiferin derivative*


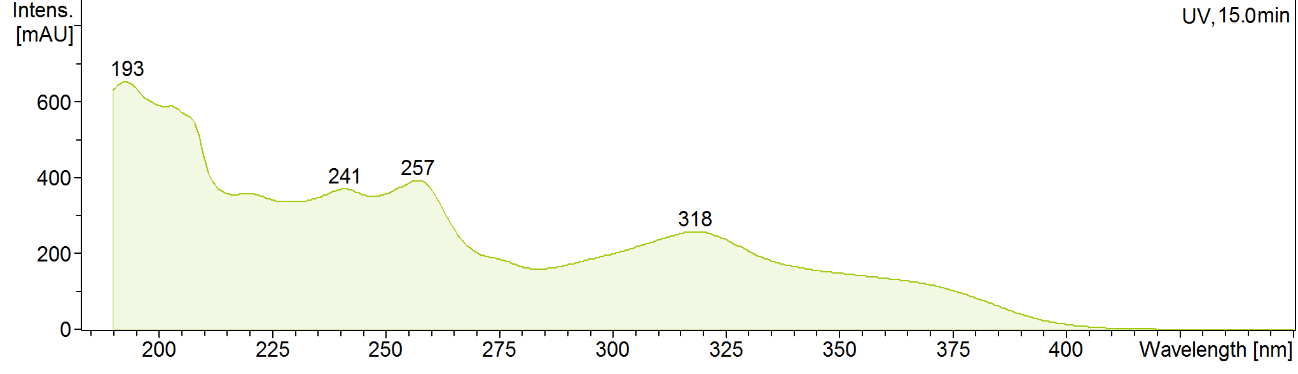


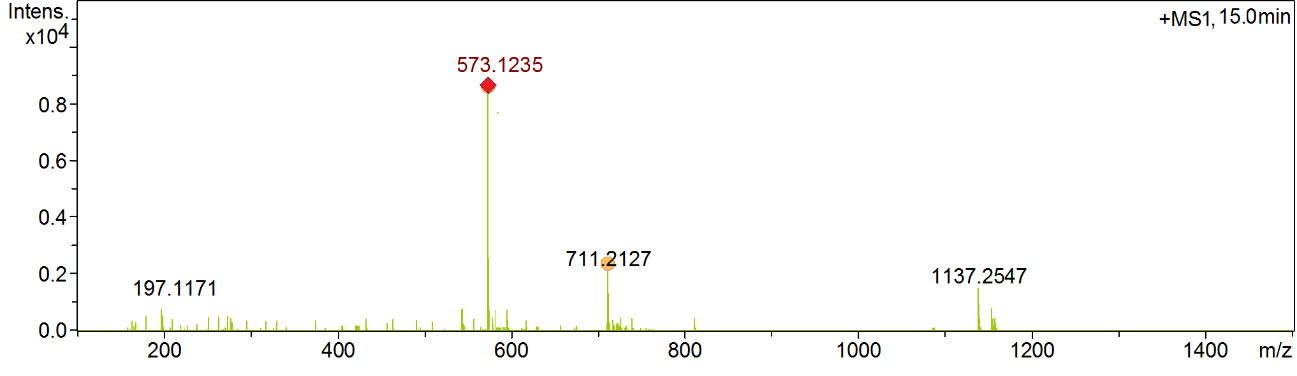


**+**

Figure 17S. MS^1^ spectrum of C-glycosyl xanthone present in the ethanolic extract of *F. formosa* leaves

Figure 16S. UV spectrum of C-glycosyl xanthone present in the ethanolic extract of *F. formosa* leaves


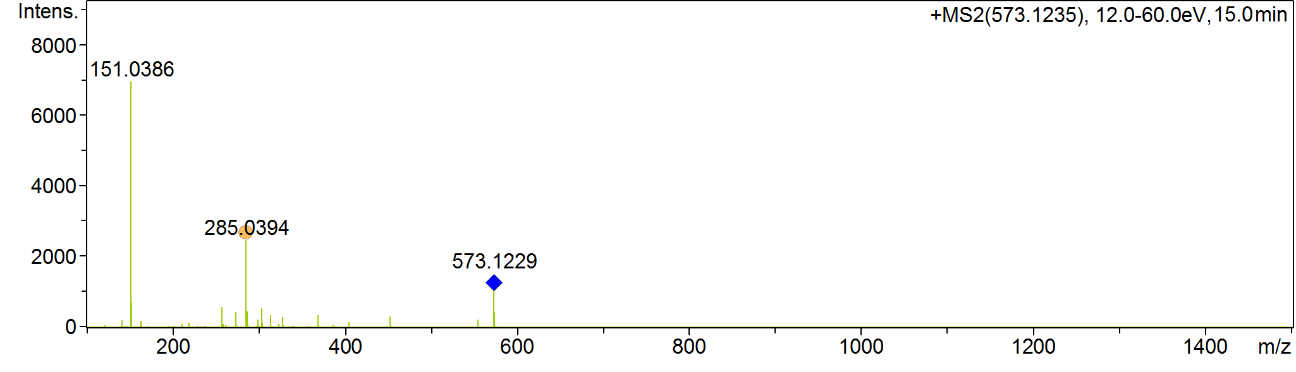


Figure 18S. MS^2^ spectrum of C-glycosyl xanthone present in the ethanolic extract of *F. formosa* leaves

**Compound (7) RT 15.1 min. MM 584 Da -** *2′-O-trans-caffeoylmangiferin*

Figure 19S. UV spectrum of C-glycosyl xanthone present in the ethanolic extract of *F. formosa* leaves


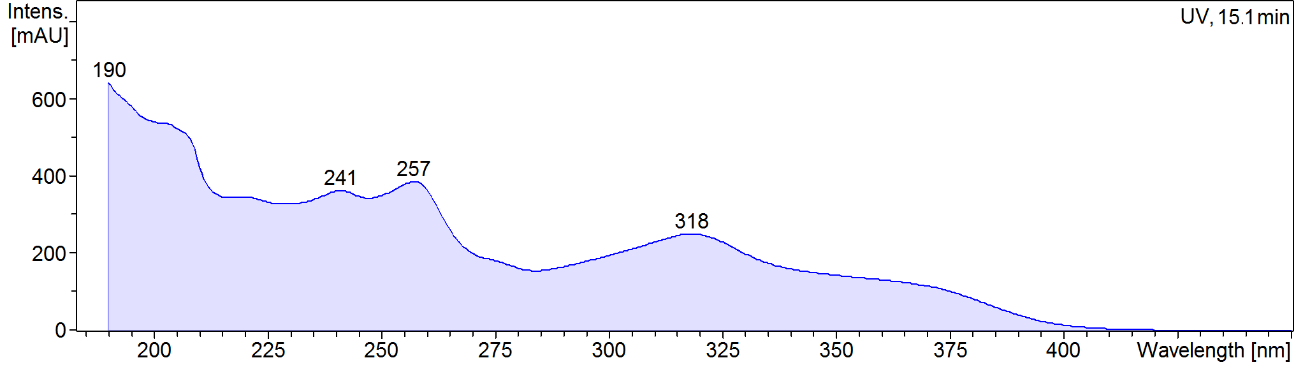


Figure 20S. MS^1^ spectrum of C-glycosyl xanthone present in the ethanolic extract of *F. formosa* leaves


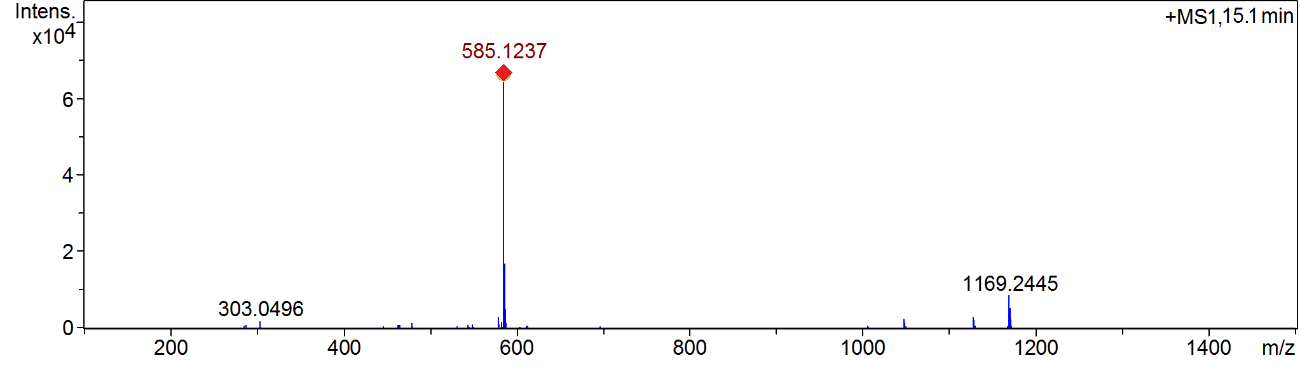


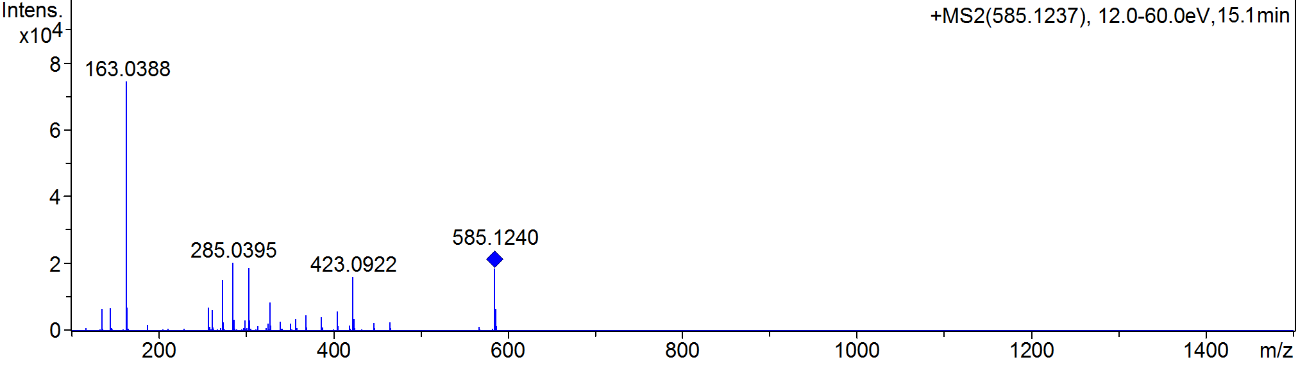


Figure 21S. MS^2^ spectrum of C-glycosyl xanthone present in the ethanolic extract of *F. formosa* leaves

**Compound (8) RT 15.2 min. MM 542 Da -** *p-Hydroxybenzoylmangiferin derivative*


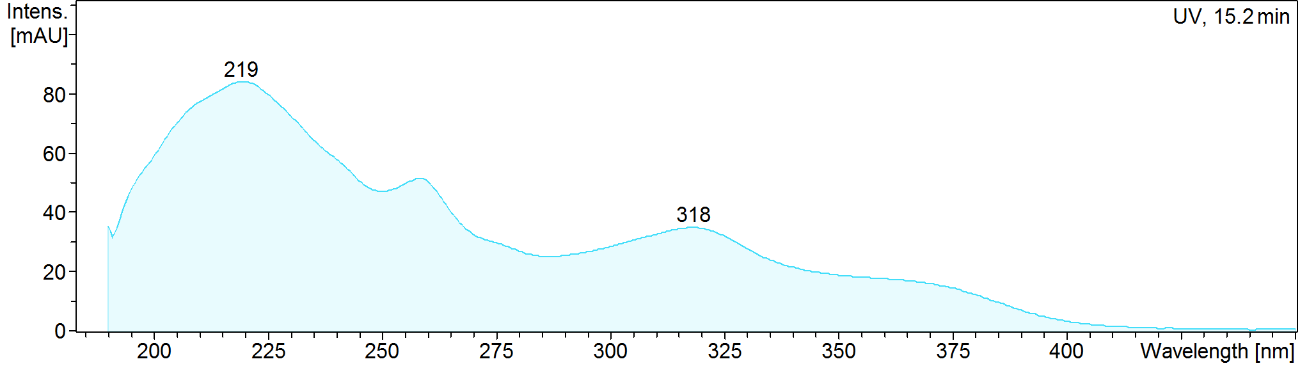


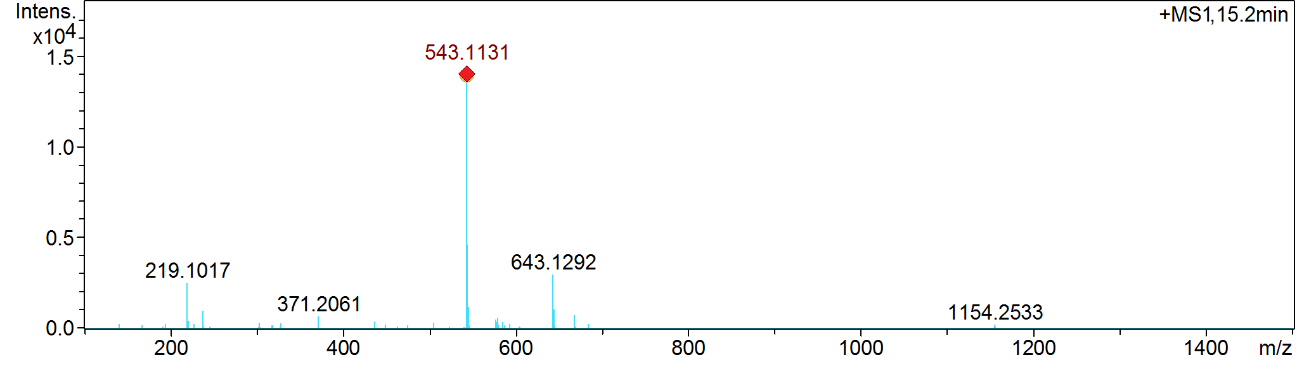


**+**

Figure 22S. UV spectrum of C-glycosyl xanthone present in the ethanolic extract of *F. formosa* leaves

Figure 23S. MS^1^ spectrum of C-glycosyl xanthone present in the ethanolic extract of *F. formosa* leaves


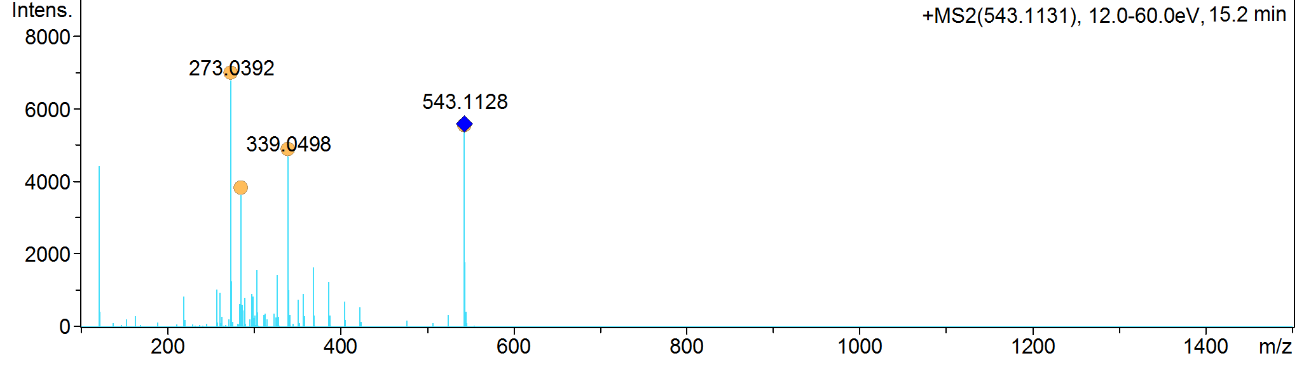


Figure 24S. MS^2^ spectrum of C-glycosyl xanthone present in the ethanolic extract of *F. formosa* leaves

**Compound (9) RT 15.4 min. MM 584 Da -** *Caffeoylmangiferin derivative*


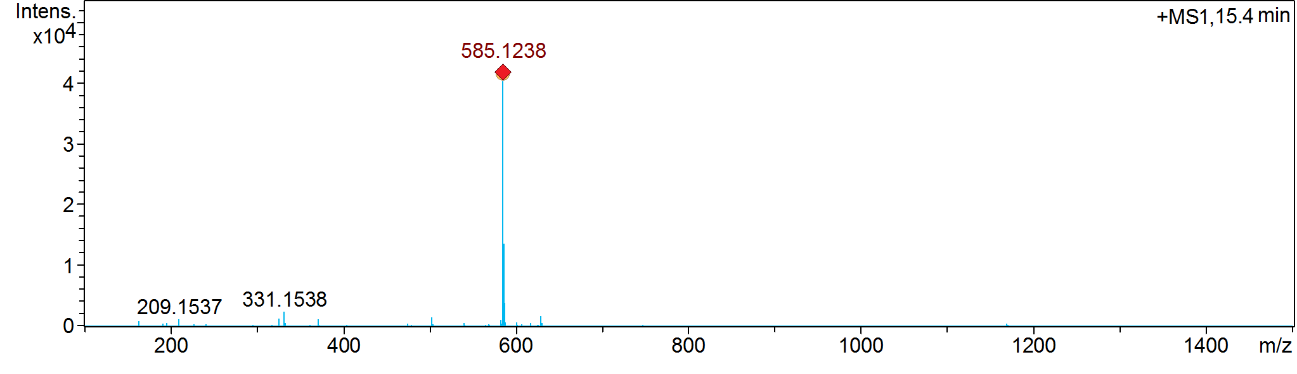

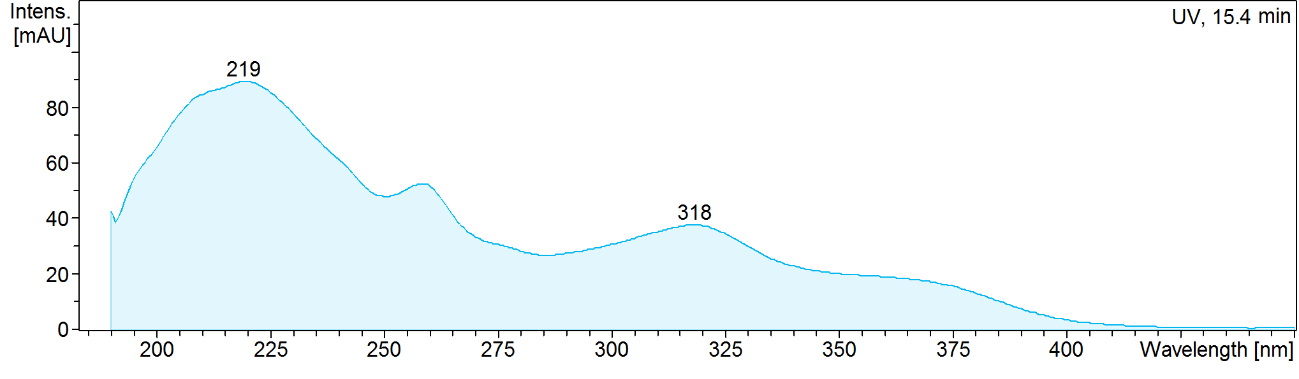


**+**

Figure 26S. MS^1^ spectrum of C-glycosyl xanthone present in the ethanolic extract of *F. formosa* leaves

Figure 25S. UV spectrum of C-glycosyl xanthone present in the ethanolic extract of *F. formosa* leaves


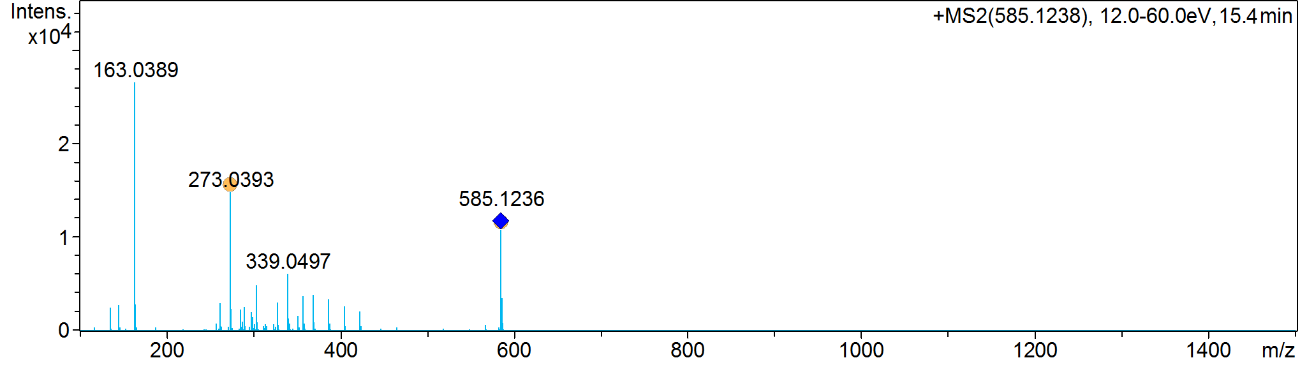


Figure 27S. MS^2^ spectrum of C-glycosyl xanthone present in the ethanolic extract of *F. formosa* leaves


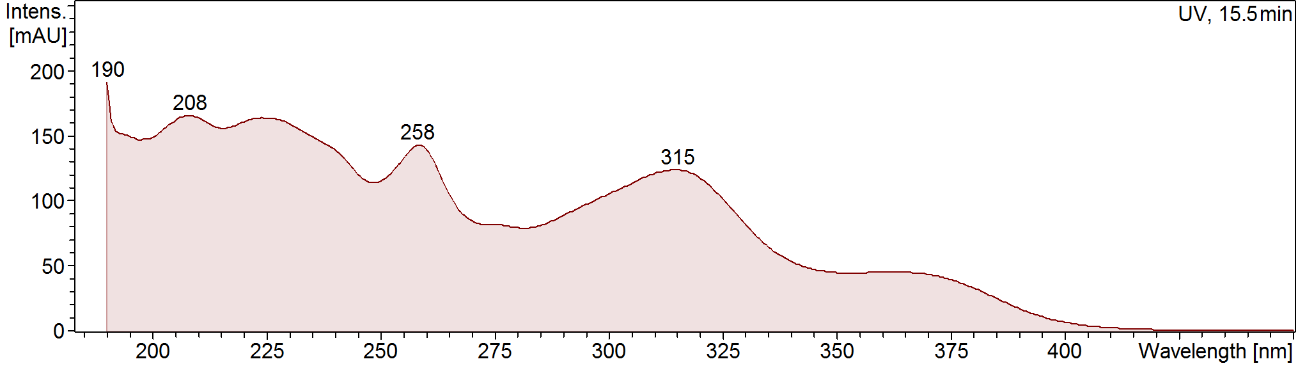
**Compound (10) RT 15.5 min. MM 528 Da -** *Mangiferin derivative*


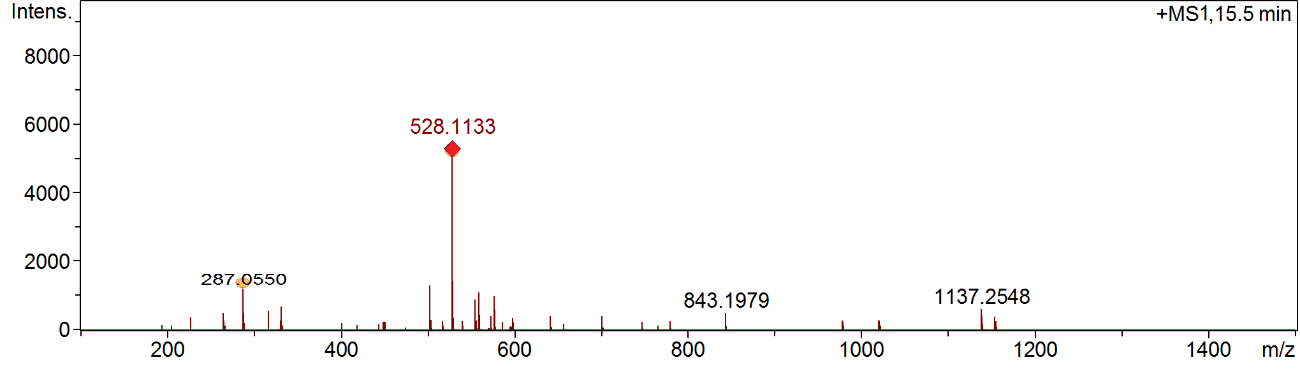


Figure 28S. UV spectrum of C-glycosyl xanthone present in the ethanolic extract of *F. formosa* leaves


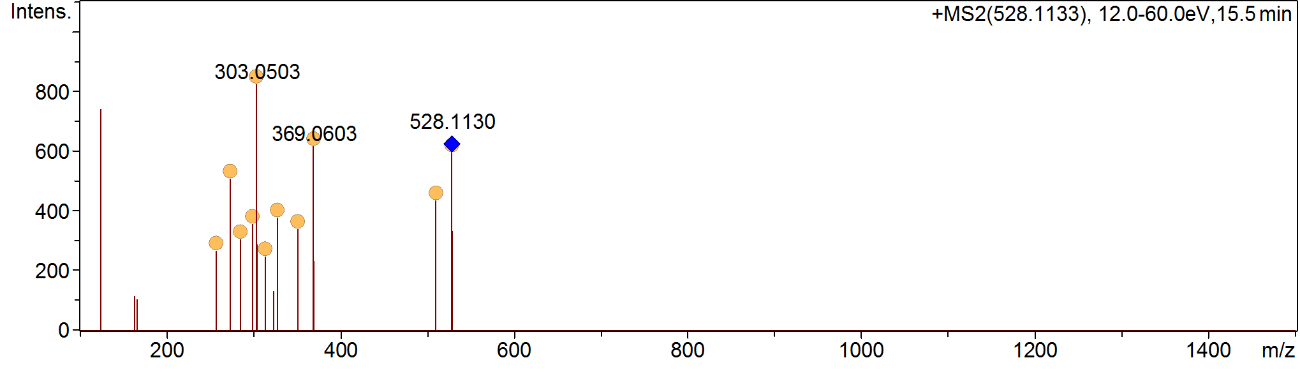


Figure 29S. MS^1^ spectrum of C-glycosyl xanthone present in the ethanolic extract of *F. formosa* leaves

Figure 30S. MS^2^ spectrum of C-glycosyl xanthone present in the ethanolic extract of *F. formosa* leaves

**Compound (11) RT 15.8 min. MM 568 Da -** *2′-O-trans-coumaroylmangiferin*


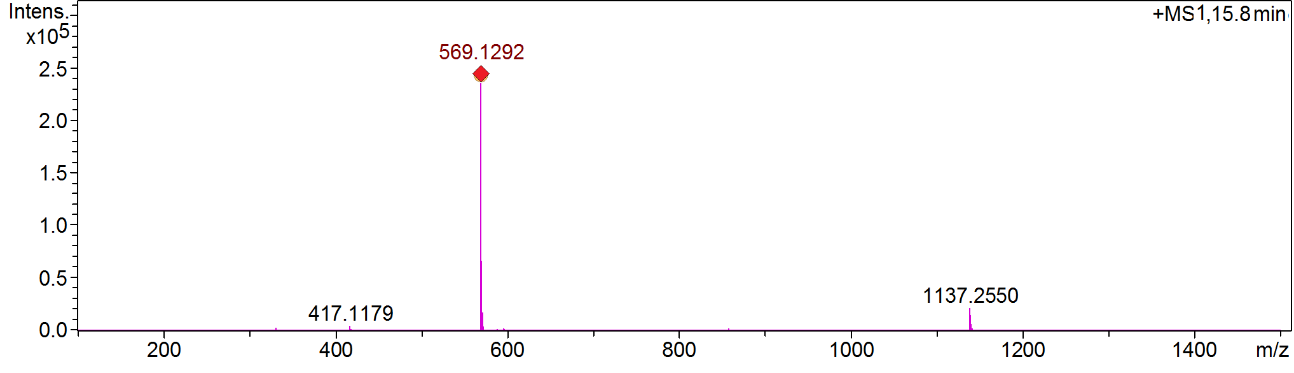

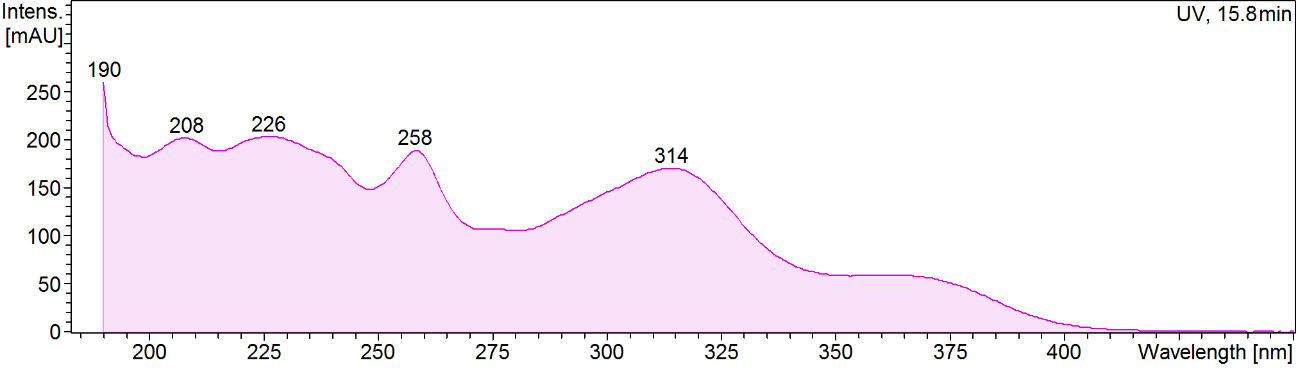


Figure 31S. UV spectrum of C-glycosyl xanthone present in the ethanolic extract of *F. formosa* leaves

Figure 32S. MS^1^ spectrum of C-glycosyl xanthone present in the ethanolic extract of *F. formosa* leaves


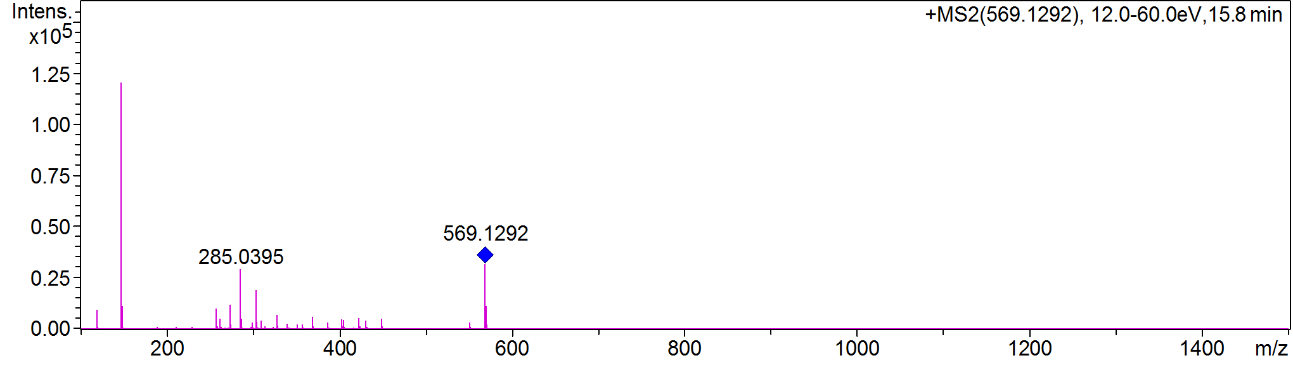


Figure 33S. MS^2^ **spectrum of C-glycosyl xanthone present in the ethanolic extract of *F. formosa* leaves**

**Compound (12) RT 15.9 min. MM 628 Da -** *Mangiferin derivative*

Figure 34S. UV spectrum of C-glycosyl xanthone present in the ethanolic extract of *F. formosa* leaves


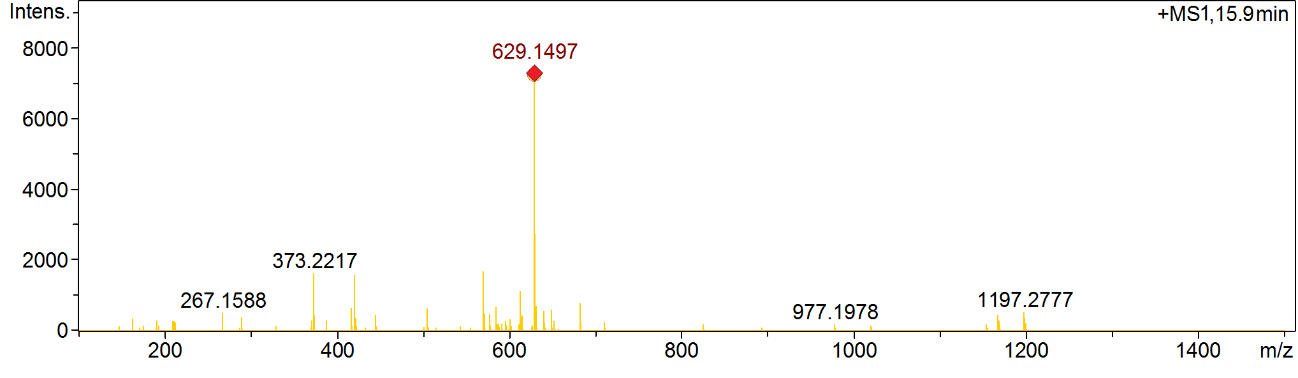

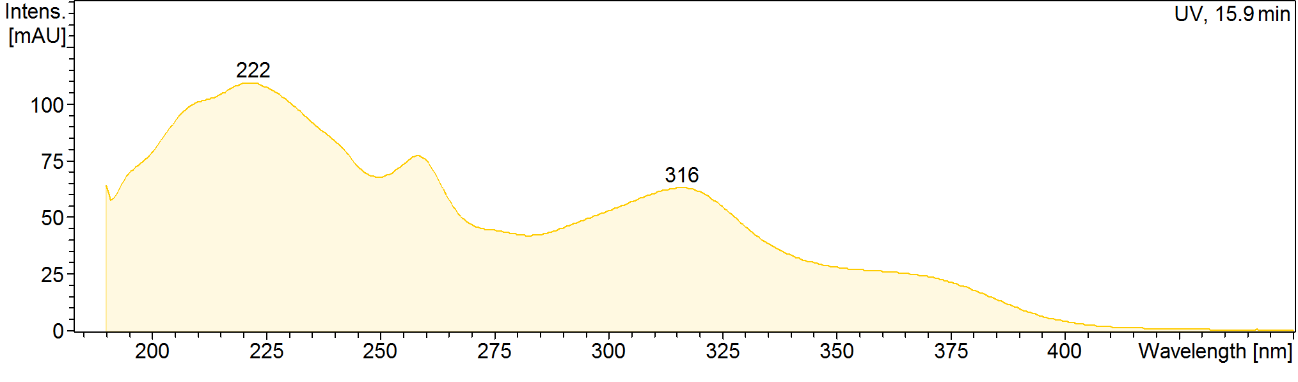


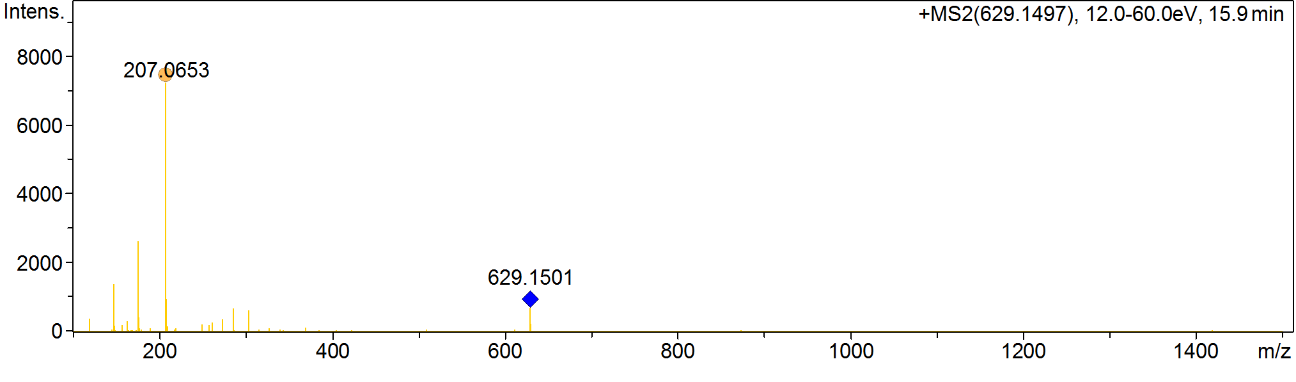


Figure 36S. MS^2^ spectrum of C-glycosyl xanthone present in the ethanolic extract of *F. formosa* leaves

Figure 35S. MS^1^ spectrum of C-glycosyl xanthone present in the ethanolic extract of *F. formosa* leaves

**Compound (13) RT 16.0 min. MM 598 Da -** *Metoxi-O-caffeoylmangiferin derivative*


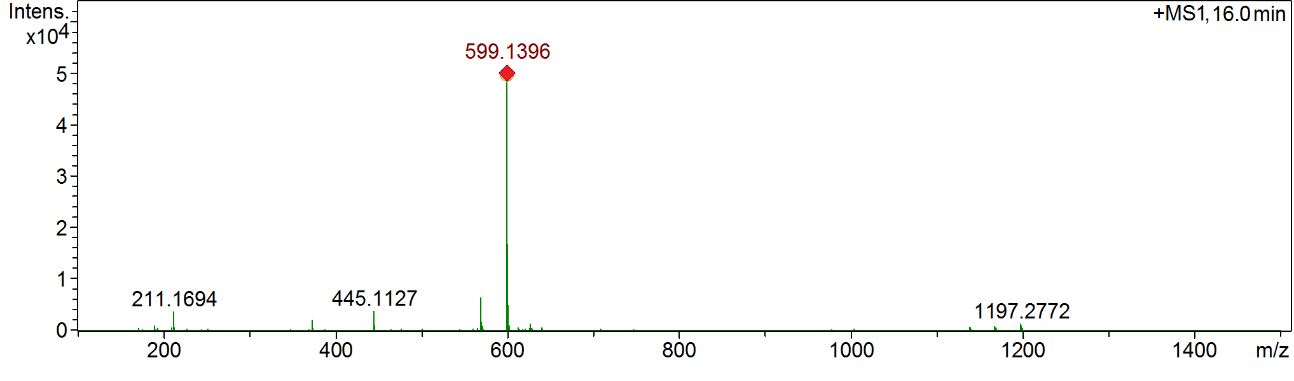

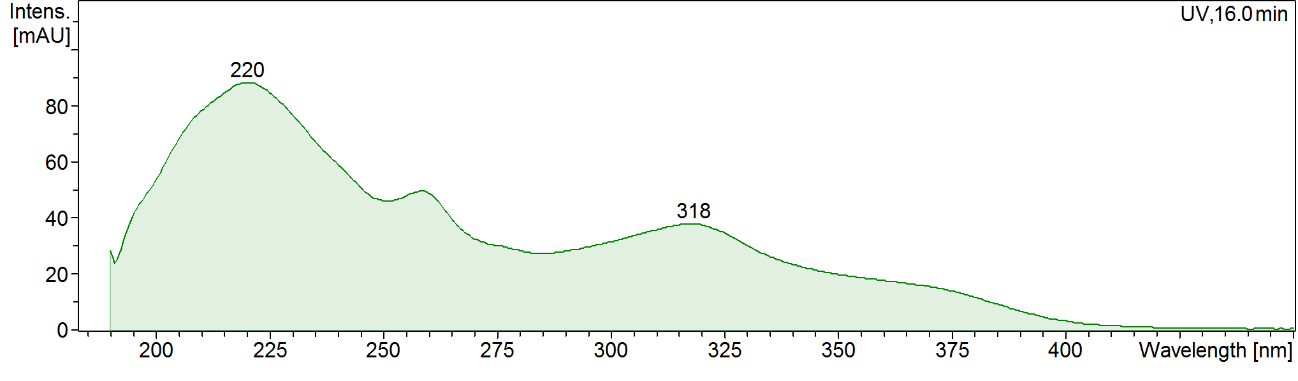


**+**

Figure 38S. MS^1^ spectrum of C-glycosyl xanthone present in the ethanolic extract of *F. formosa* leaves

Figure 37S. UV spectrum of C-glycosyl xanthone present in the ethanolic extract of *F. formosa* leaves

Figure 39S. MS^2^ spectrum of C-glycosyl xanthone present in the ethanolic extract of *F. formosa* leaves


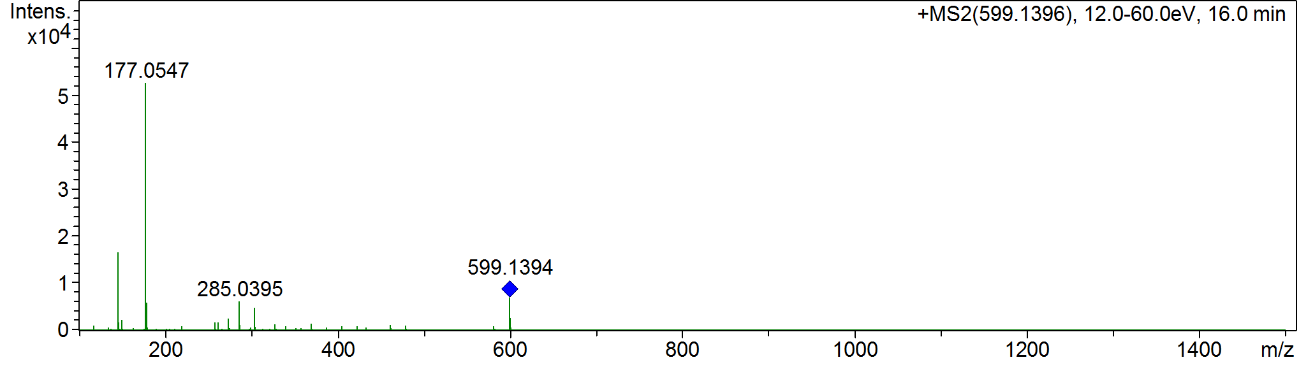


**Compound (14) RT 16.3 min. MM 568 Da -** *Coumaroylmangiferin derivative*


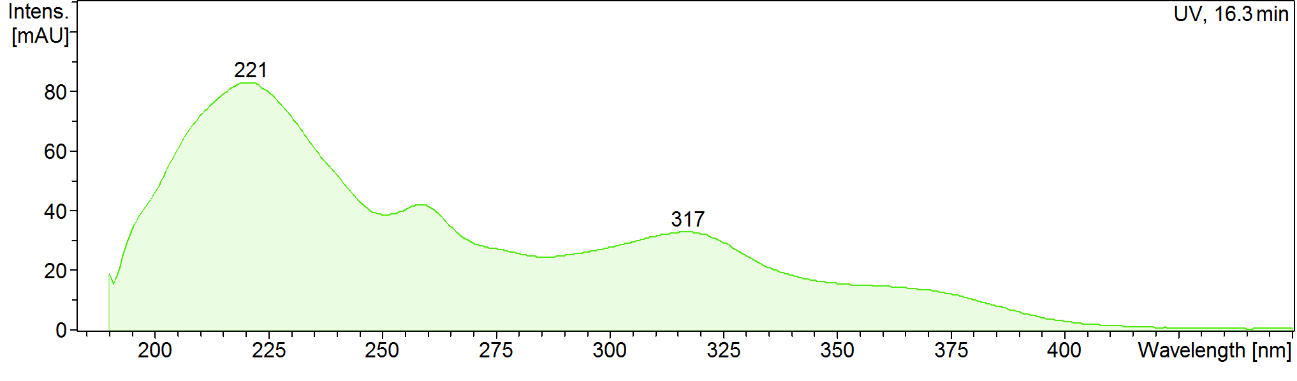


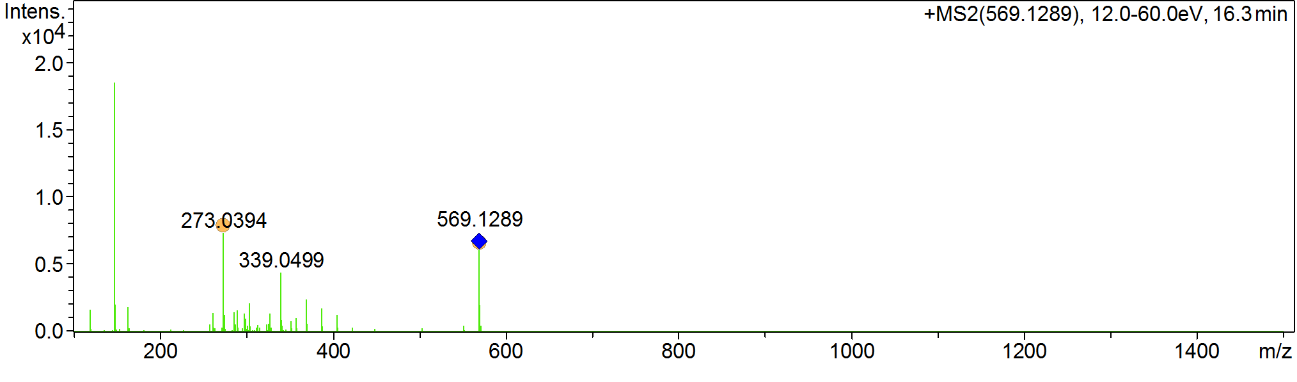

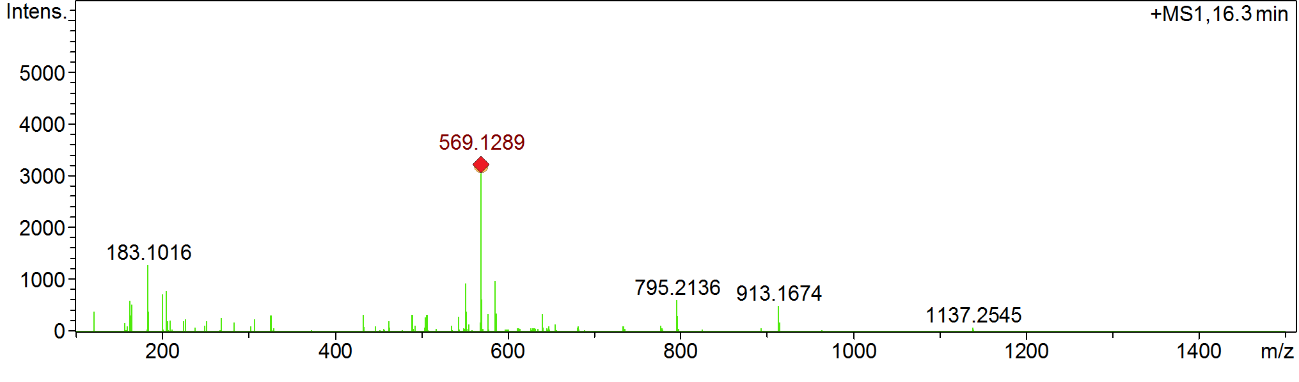


**+**

Figure 42S. MS^2^ spectrum of C-glycosyl xanthone present in the ethanolic extract of *F. formosa* leaves

Figure 41S. MS^1^ spectrum of C-glycosyl xanthone present in the ethanolic extract of *F. formosa* leaves

Figure 40S. UV spectrum of C-glycosyl xanthone present in the ethanolic extract of *F. formosa* leaves

**Compound (15) RT 16.4 min. MM 568 Da -** *Coumaroylmangiferin derivative*


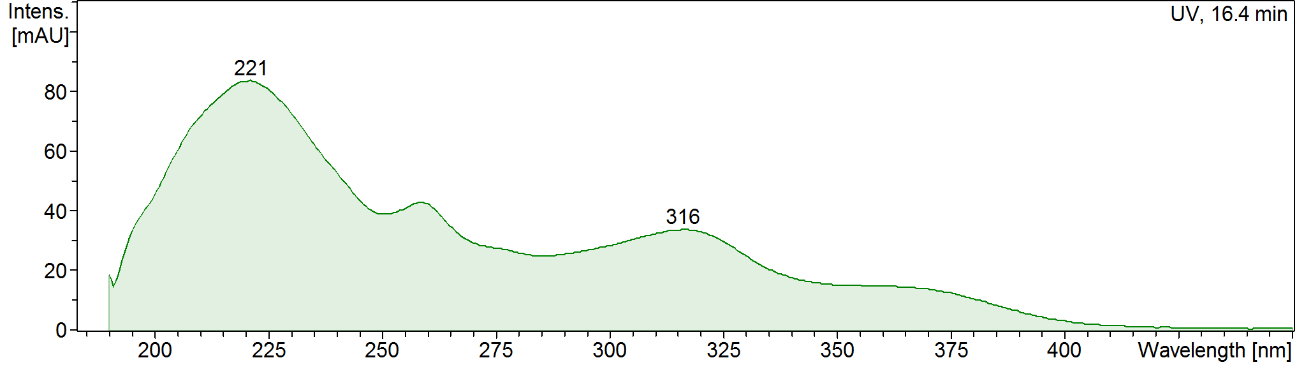


Figure 43S. UV spectrum of C-glycosyl xanthone present in the ethanolic extract of *F. formosa* leaves


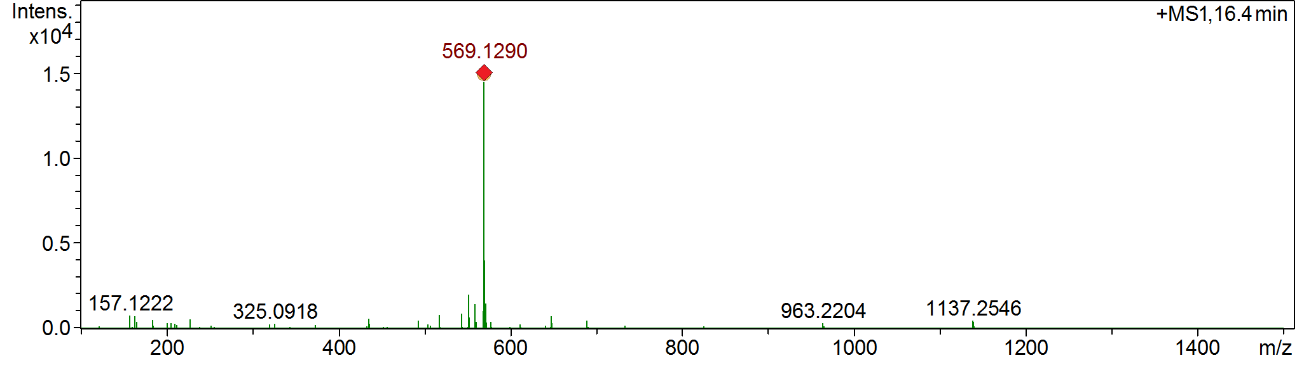


**+**

Figure 44S. MS^1^ spectrum of C-glycosyl xanthone present in the ethanolic extract of *F. formosa* leaves


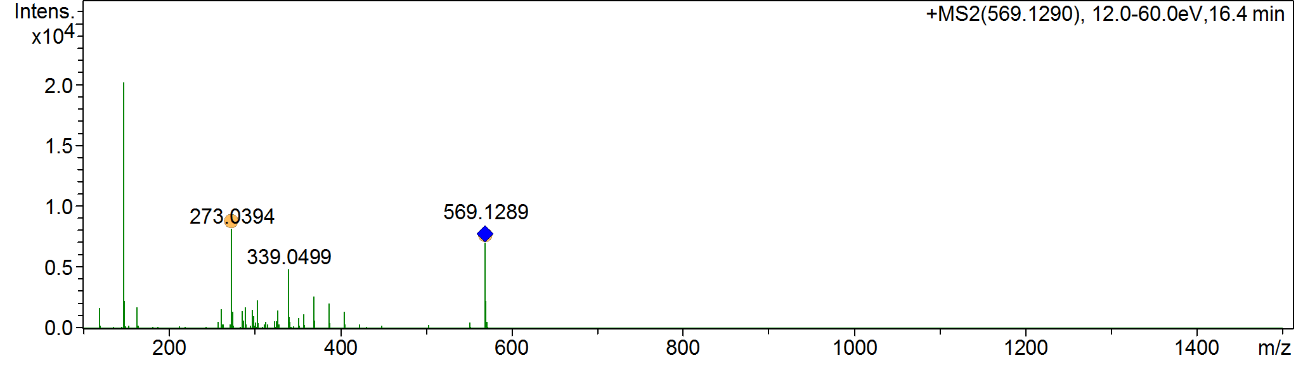


Figure 45S. MS^2^ spectrum of C-glycosyl xanthone present in the ethanolic extract of *F. formosa* leaves

**Compound (16) RT 16.6 min. MM 584 Da -** *Caffeoylmangiferin derivative*


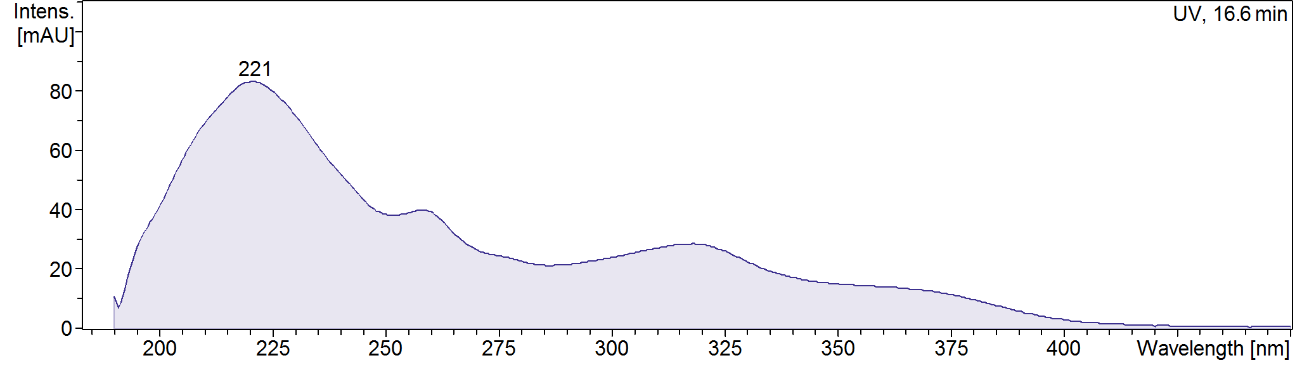


Figure 46S. UV spectrum of C-glycosyl xanthone present in the ethanolic extract of *F. formosa* leaves


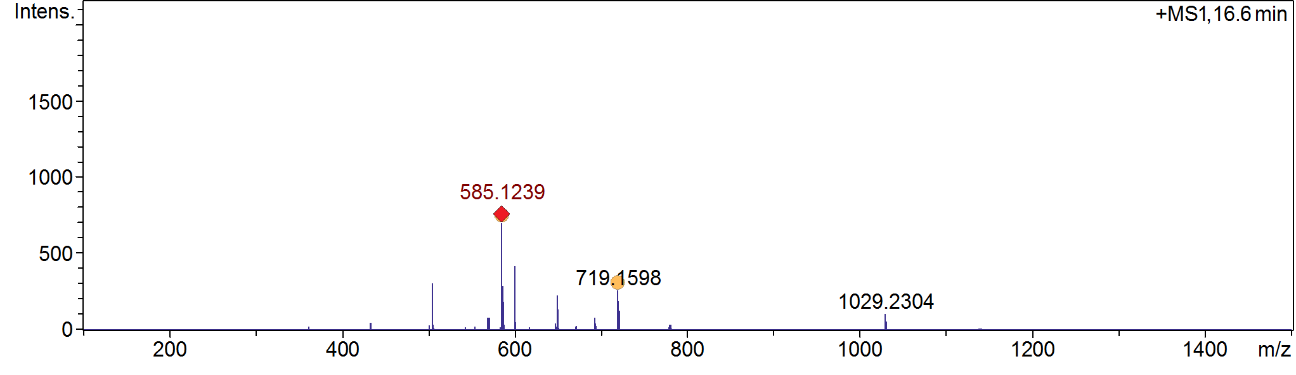


**+**

Figure 47S. MS^1^ spectrum of C-glycosyl xanthone present in the ethanolic extract of *F. formosa* leaves


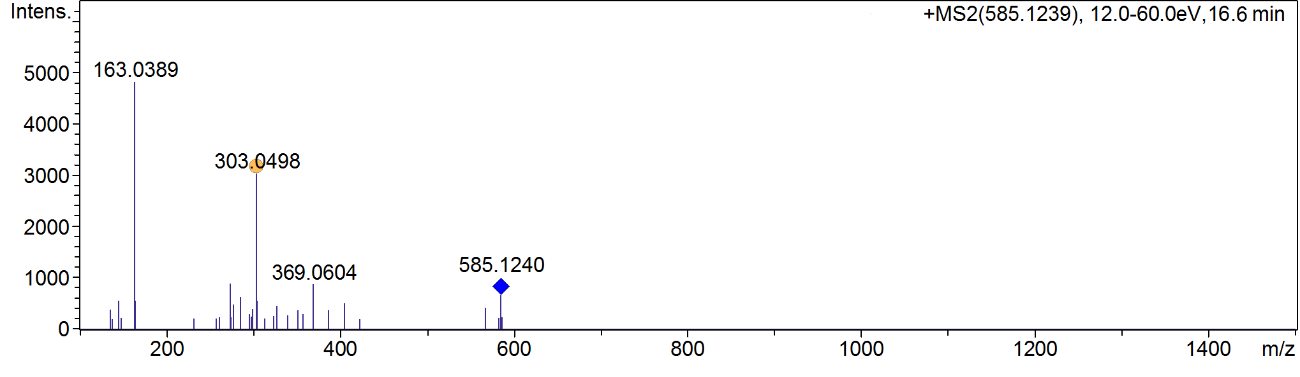


Figure 48S. MS^2^ spectrum of C-glycosyl xanthone present in the ethanolic extract of *F. formosa* leaves


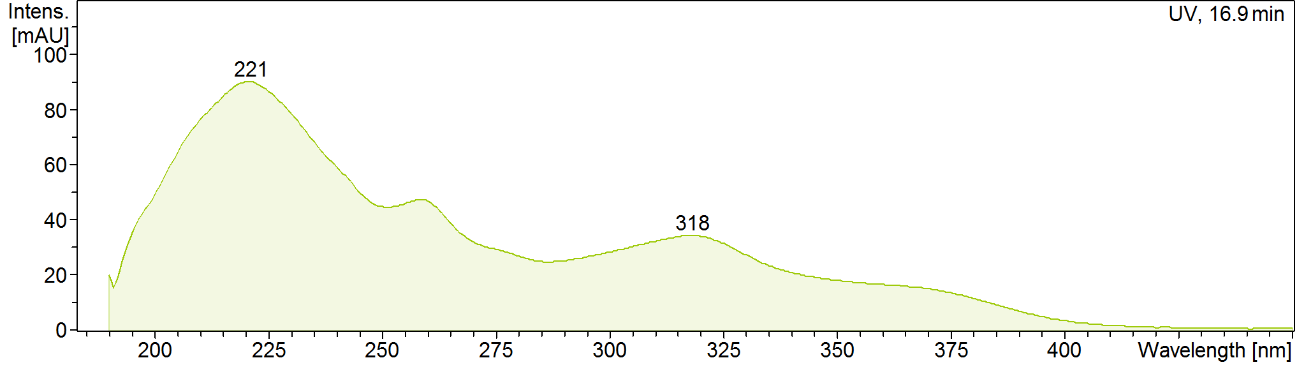
**Compound (17) RT 16.9 min. MM 526 Da -** *Benzoylmangiferin derivative*

Figure 49S. UV spectrum of C-glycosyl xanthone present in the ethanolic extract of *F. formosa* leaves


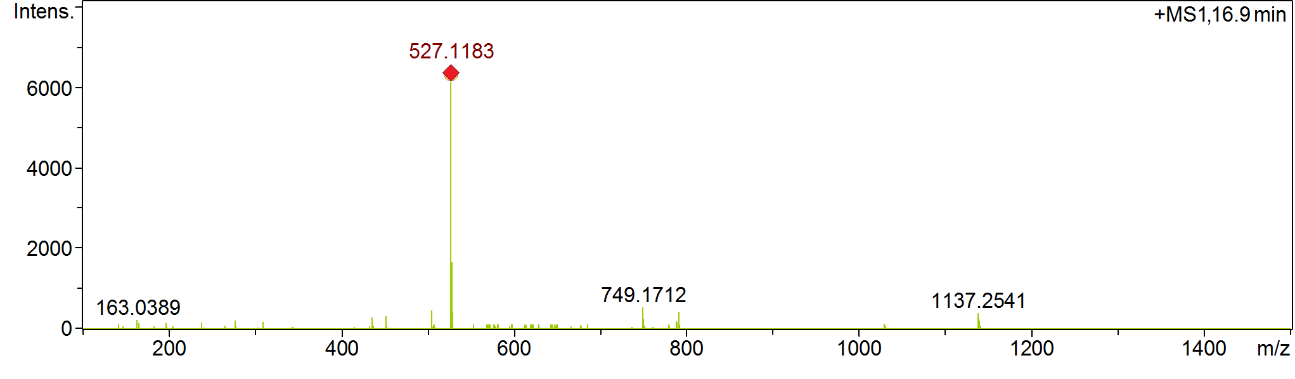


**+**

Figure 50S. MS^1^ spectrum of C-glycosyl xanthone present in the ethanolic extract of *F. formosa* leaves


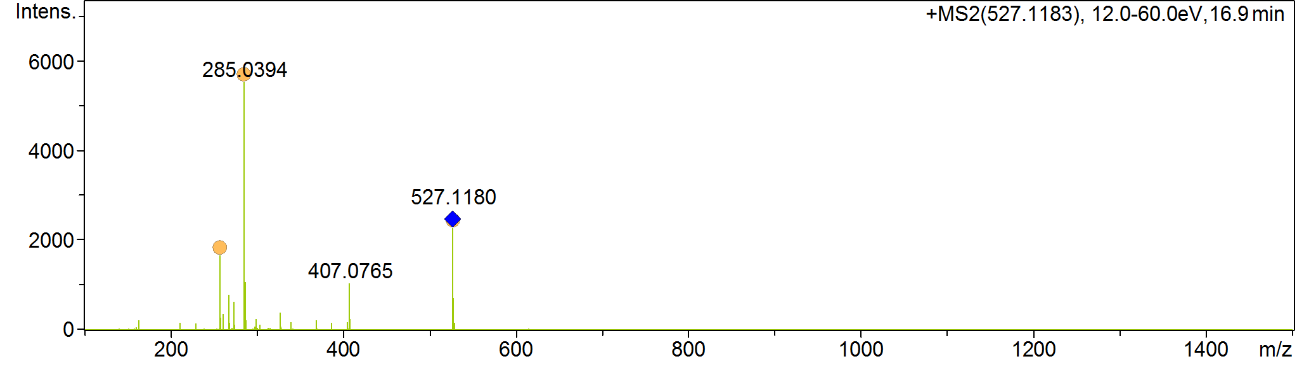


Figure 51S. MS^2^ spectrum of C-glycosyl xanthone present in the ethanolic extract of *F. formosa* leaves

**Compound (18) RT 17.1 min. MM 584 Da -** *Caffeoylmangiferin derivative*


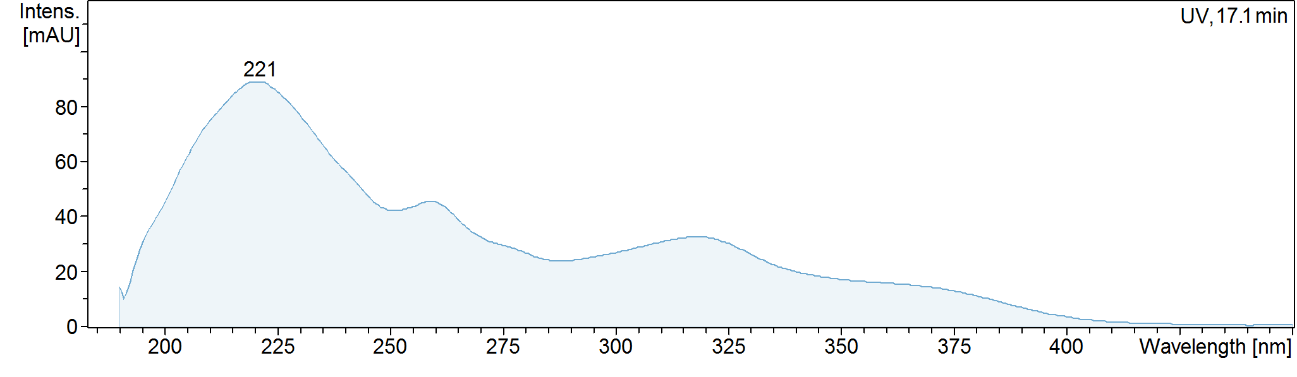


Figure 52S. UV spectrum of C-glycosyl xanthone present in the ethanolic extract of *F. formosa* leaves


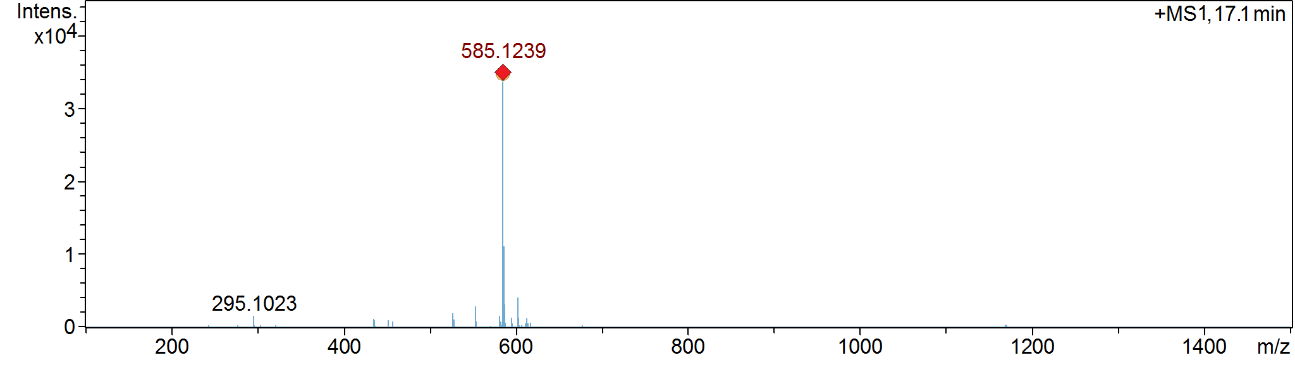


**+**

Figure 53S. MS^1^ spectrum of C-glycosyl xanthone present in the ethanolic extract of *F. formosa* leaves


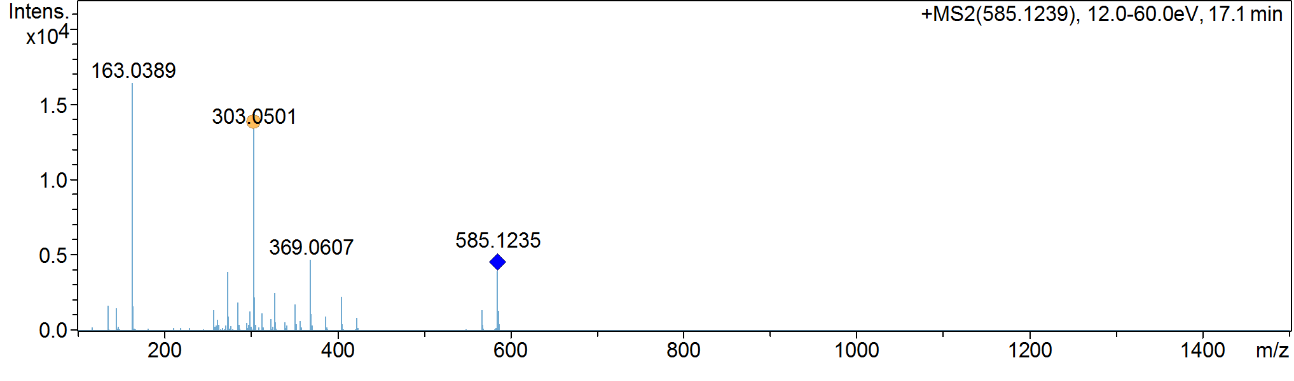


Figure 54S. MS^2^ spectrum of C-glycosyl xanthone present in the ethanolic extract of *F. formosa* leaves

**Compound (19) RT 17.4 min. MM 542 Da -** *p-Hydroxybenzoylmangiferin derivative*


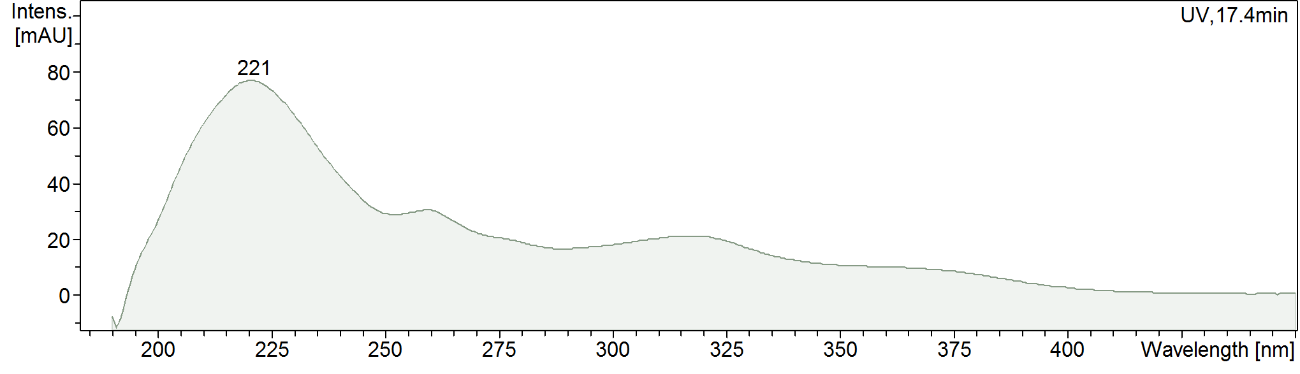


Figure 55S. UV spectrum of C-glycosyl xanthone present in the ethanolic extract of *F. formosa* leaves


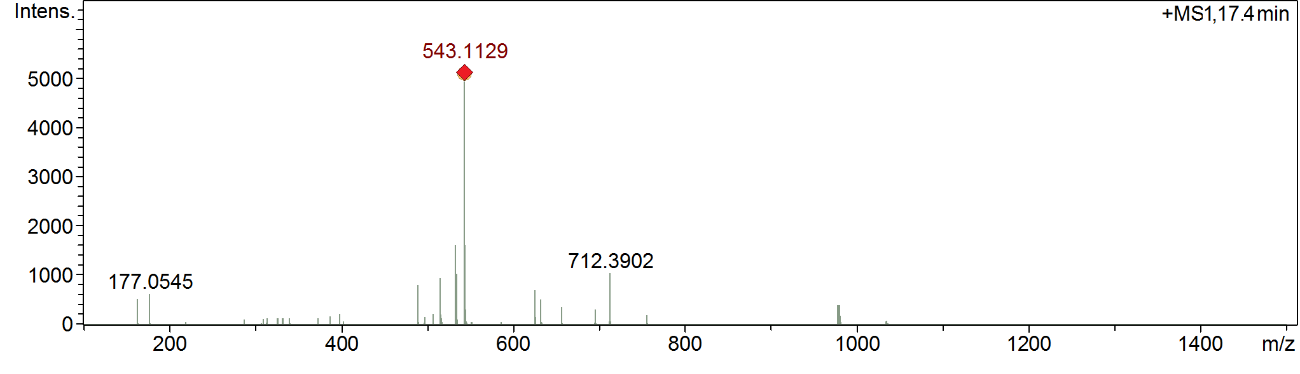


**+**

Figure 56S. MS^1^ spectrum of C-glycosyl xanthone present in the ethanolic extract of *F. formosa* leaves


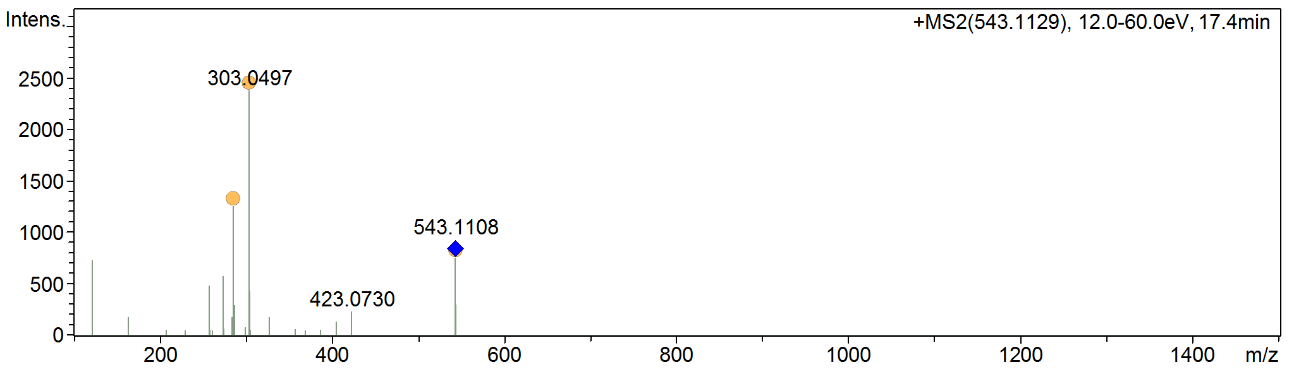


Figure 57S. MS^2^ spectrum of C-glycosyl xanthone present in the ethanolic extract of *F. formosa* leaves

**Compound (20) RT 17.5 min. MM 552 Da -** *Cinnamoylmangiferin derivative*


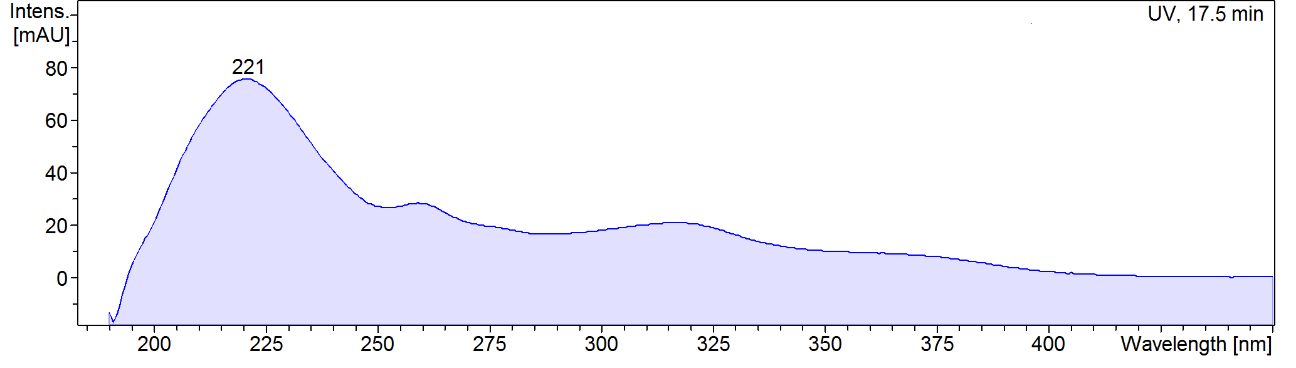


Figure 58S. UV spectrum of C-glycosyl xanthone present in the ethanolic extract of *F. formosa* leaves


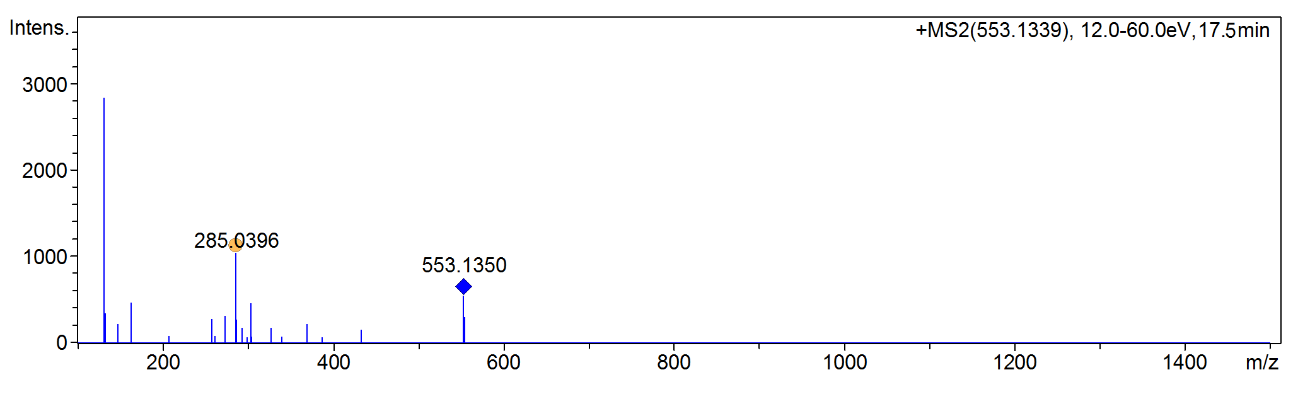

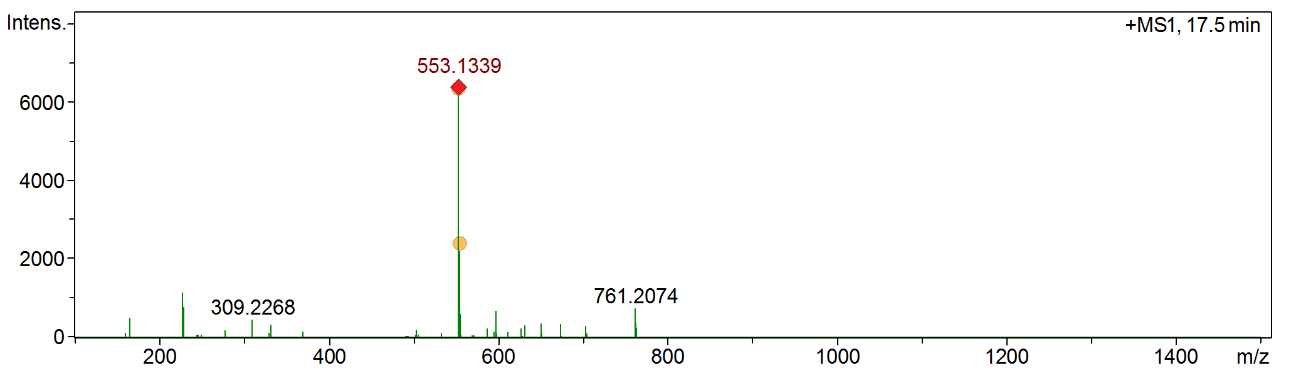


**+**

Figure 60S. MS^2^ spectrum of C-glycosyl xanthone present in the ethanolic extract of *F. formosa* leaves

Figure 59S. MS^1^ spectrum of C-glycosyl xanthone present in the ethanolic extract of *F. formosa* leaves


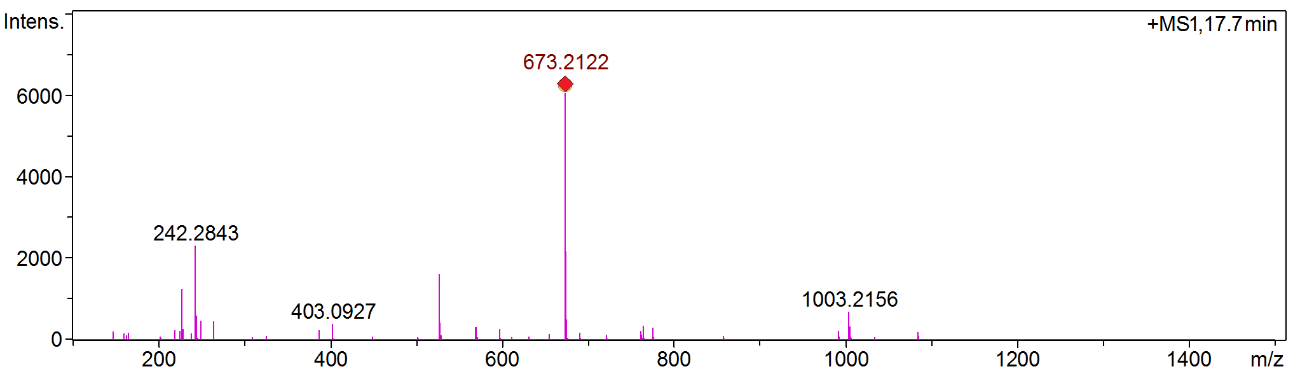

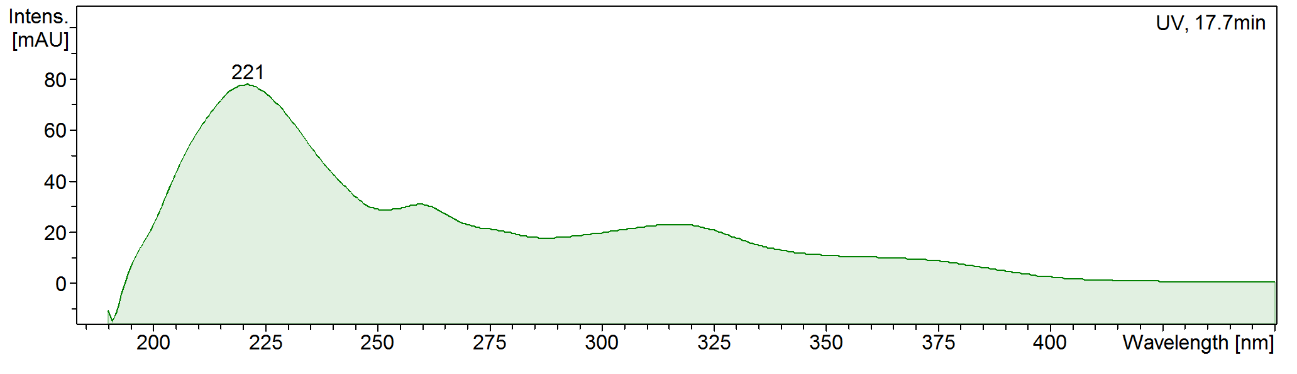
**Compound (21) RT 17.7 min. MM 672 Da -** *Mangiferin derivative*

Figure 62S. MS^1^ spectrum of C-glycosyl xanthone present in the ethanolic extract of *F. formosa* leaves

Figure 61S. UV spectrum of C-glycosyl xanthone present in the ethanolic extract of *F. formosa* leaves

Figure 63S. MS^2^ spectrum of C-glycosyl xanthone present in the ethanolic extract of *F. formosa* leaves


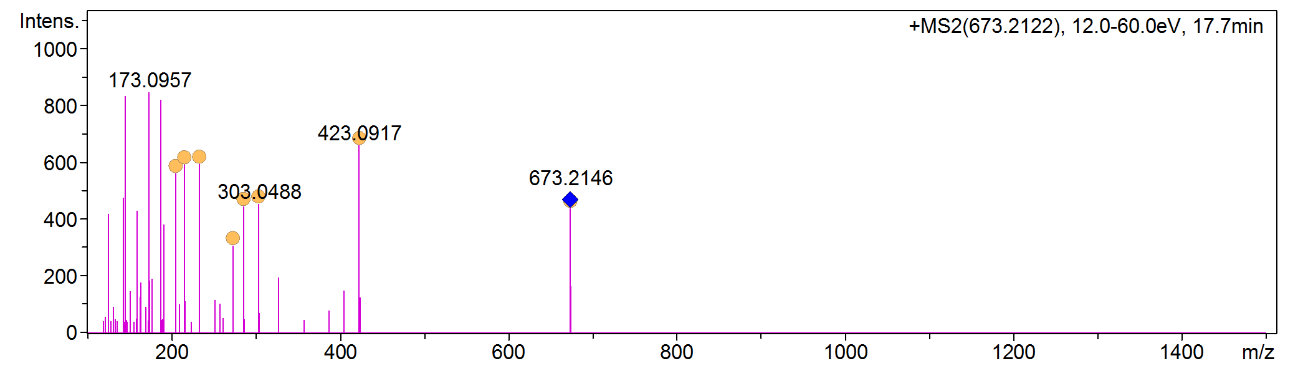


**Compound (22) RT 17.7 min. MM 568 Da -** *Coumaroylmangiferin derivative*


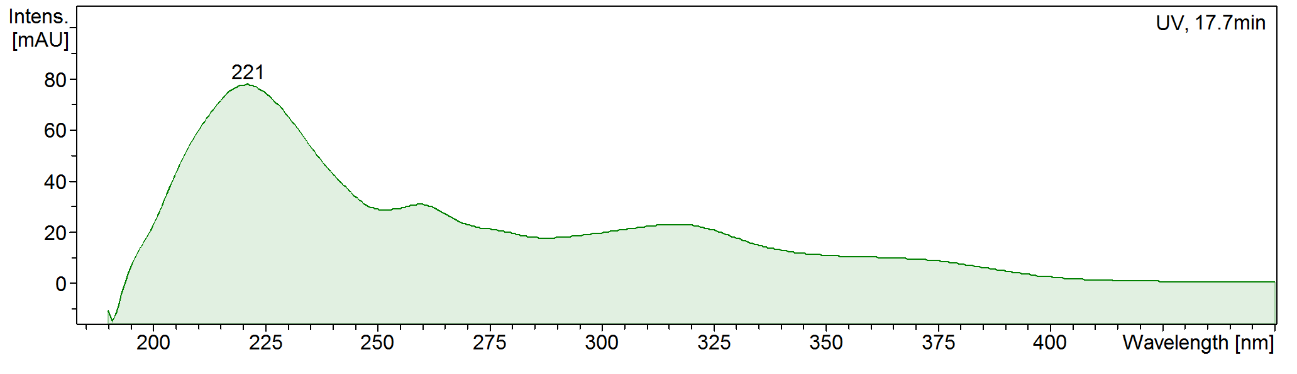


Figure 64S. UV spectrum of C-glycosyl xanthone present in the ethanolic extract of *F. formosa* leaves

**+**

Figure 65S. MS^1^ spectrum of C-glycosyl xanthone present in the ethanolic extract of *F. formosa* leaves


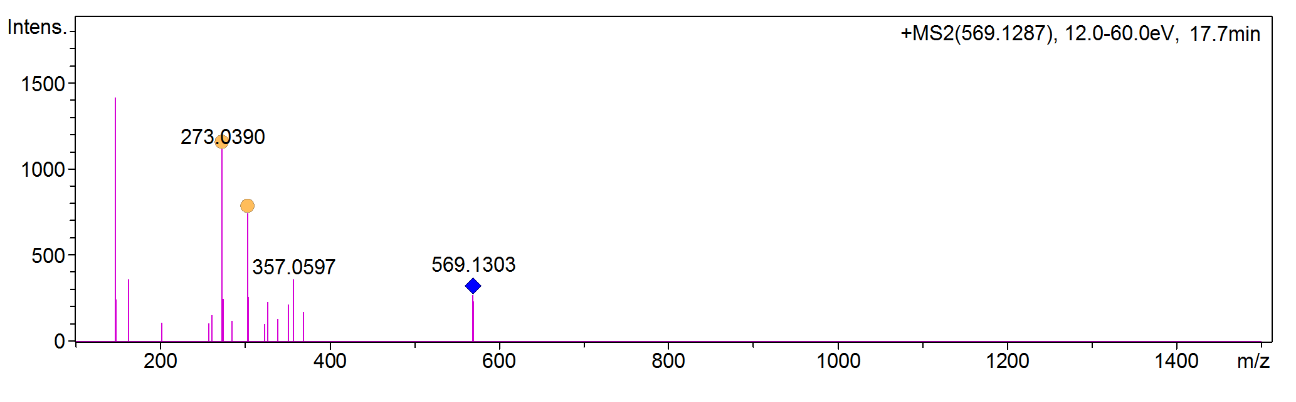

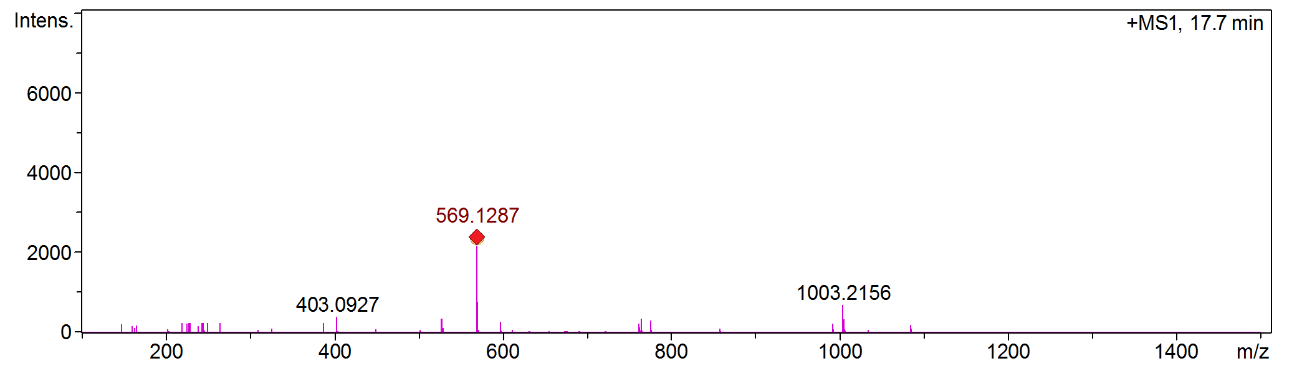


Figure 66S. MS^2^ spectrum of C-glycosyl xanthone present in the ethanolic extract of *F. formosa* leaves

**Compound (23) RT 17.8 min. MM 542 Da -** *p-Hydroxybenzoylmangiferin derivative*


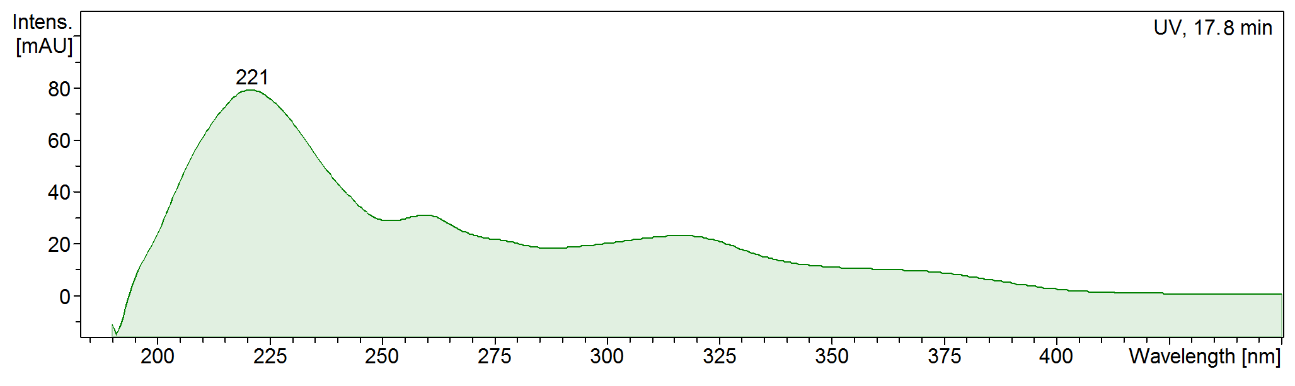


Figure 67S. UV spectrum of C-glycosyl xanthone present in the ethanolic extract of *F. formosa* leaves


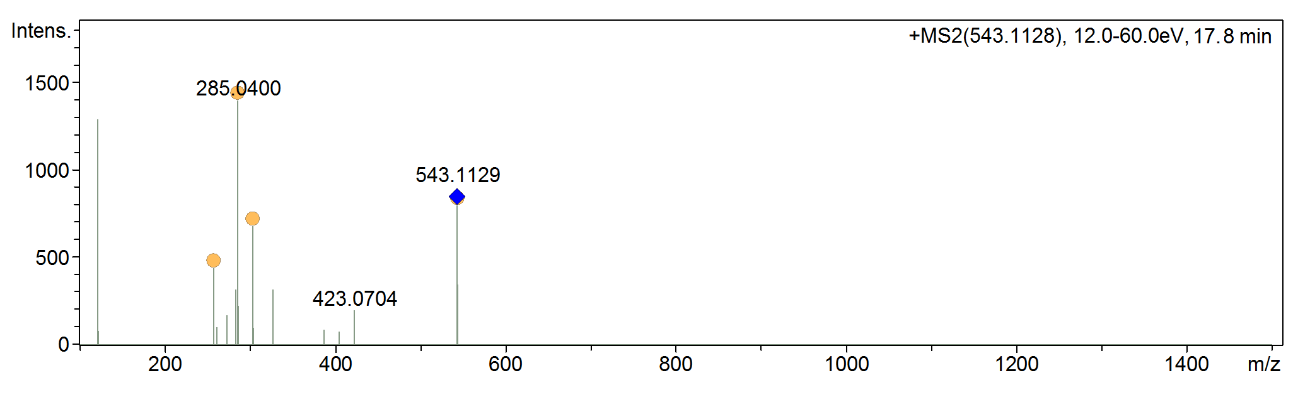

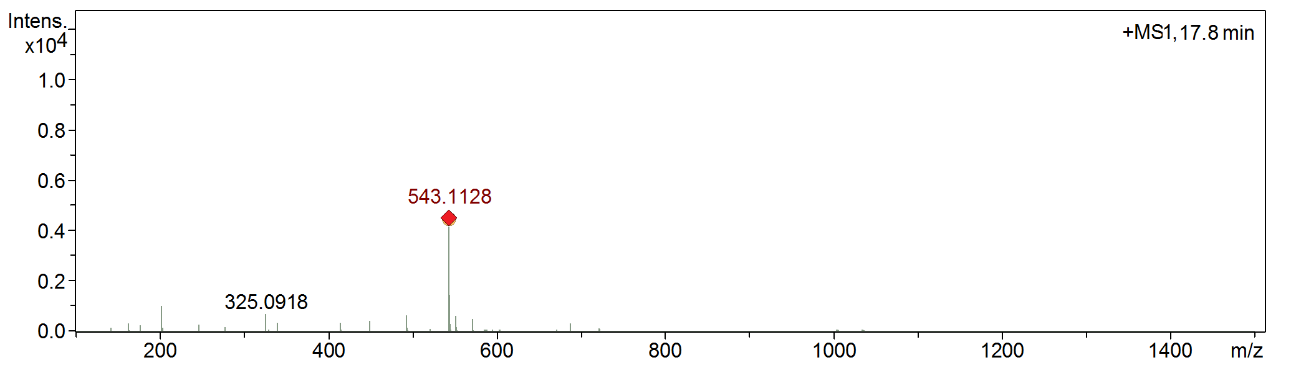


**+**

Figure 69S. MS^2^ spectrum of C-glycosyl xanthone present in the ethanolic extract of *F. formosa* leaves

Figure 68S. MS^1^ spectrum of C-glycosyl xanthone present in the ethanolic extract of *F. formosa* leaves

**Compound (24) RT 17.9 min. MM 568 Da -** *Caffeoylmangiferin derivative*


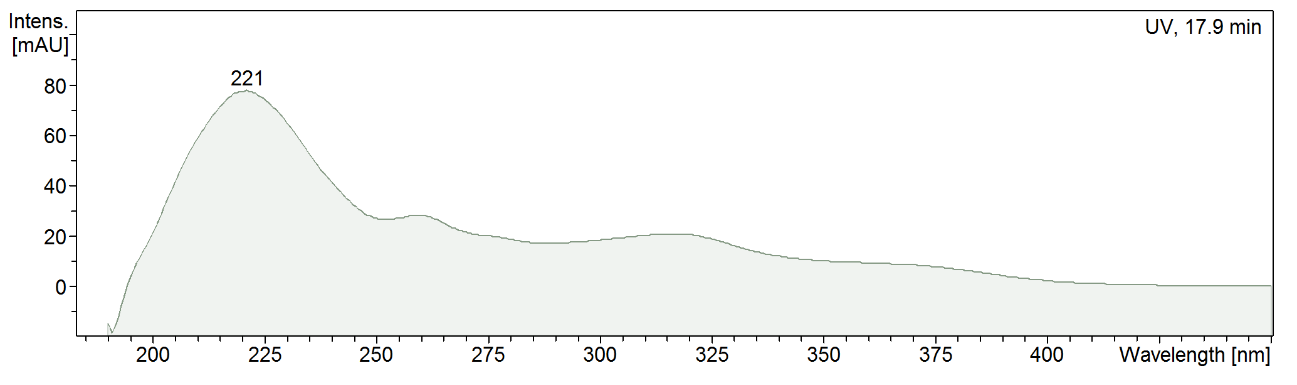


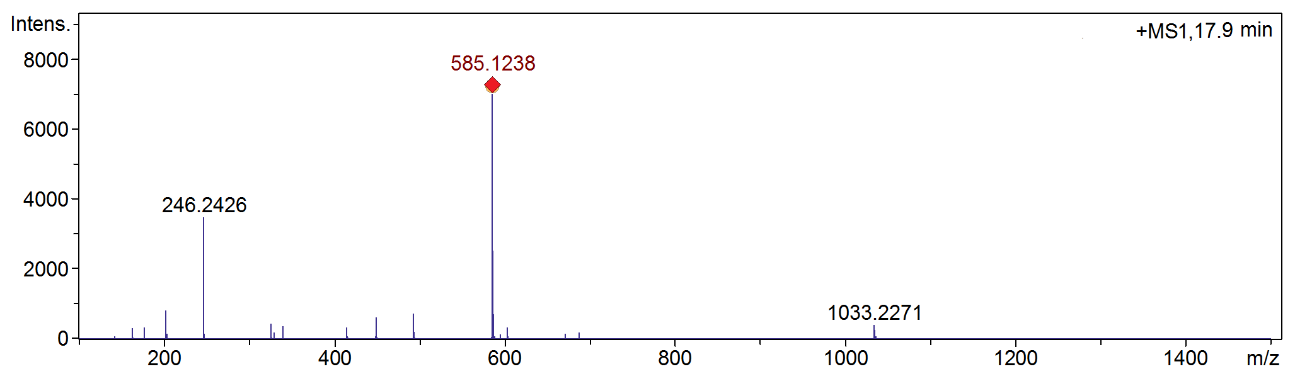


**+**

Figure 71S. MS^1^ spectrum of C-glycosyl xanthone present in the ethanolic extract of *F. formosa* leaves

Figure 70S. UV spectrum of C-glycosyl xanthone present in the ethanolic extract of *F. formosa* leaves

Figure 72S. MS^2^ spectrum of C-glycosyl xanthone present in the ethanolic extract of *F. formosa* leaves


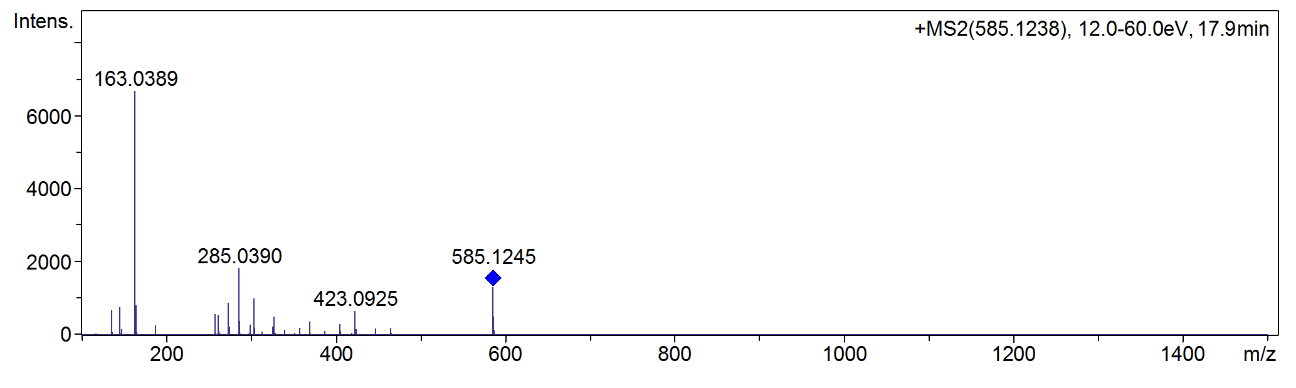


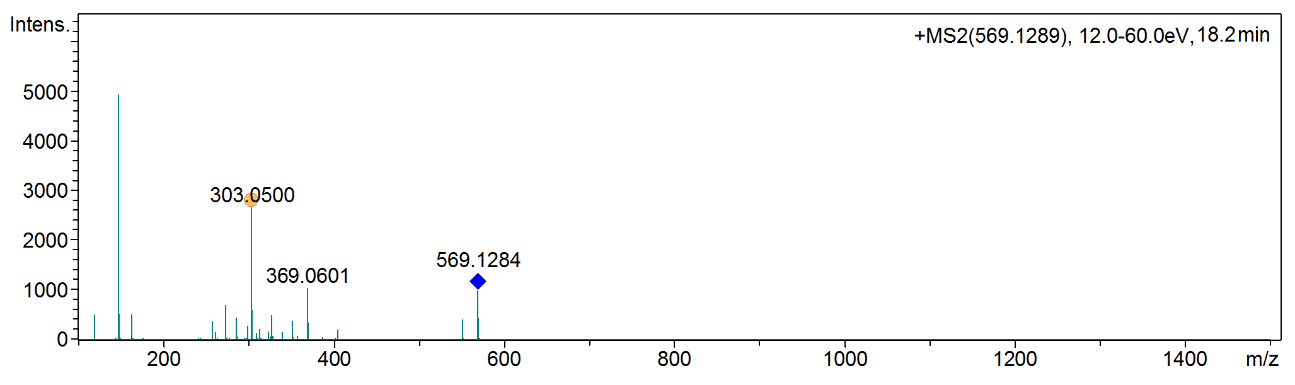

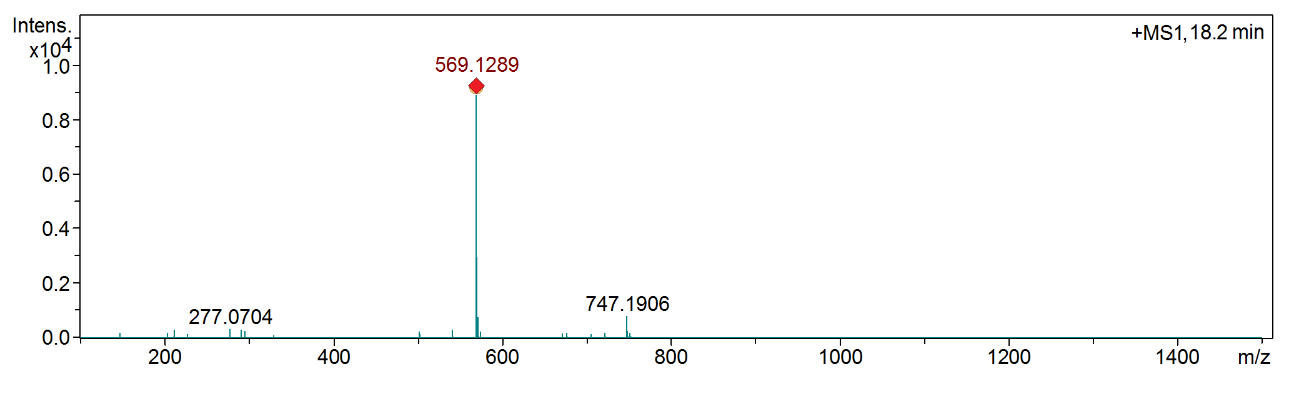

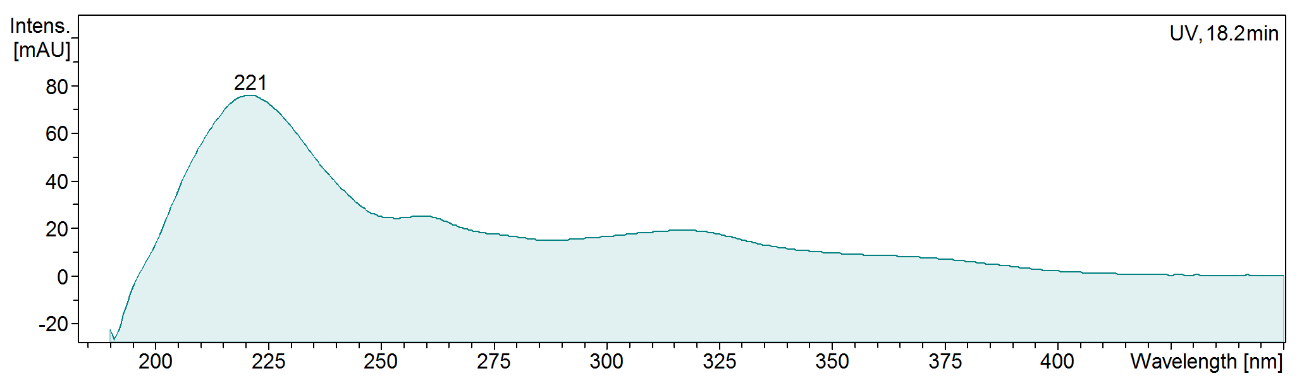
**Compound (25) RT 18.2 min. MM 568 Da -** *Coumaroylmangiferin derivative*

**+**

Figure 75S. MS^2^ spectrum of C-glycosyl xanthone present in the ethanolic extract of *F. formosa* leaves

Figure 74S. MS^1^ spectrum of C-glycosyl xanthone present in the ethanolic extract of *F. formosa* leaves

Figure 73S. UV spectrum of C-glycosyl xanthone present in the ethanolic extract of *F. formosa* leaves

**Compound (26) RT 18.6 min. MM 568 Da -** *2′-O-trans-cinnamoylmangiferin*


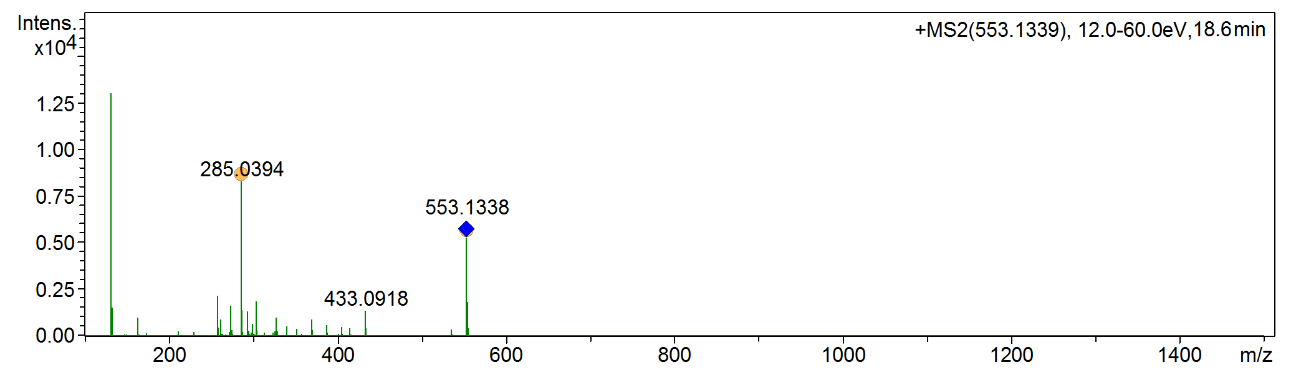

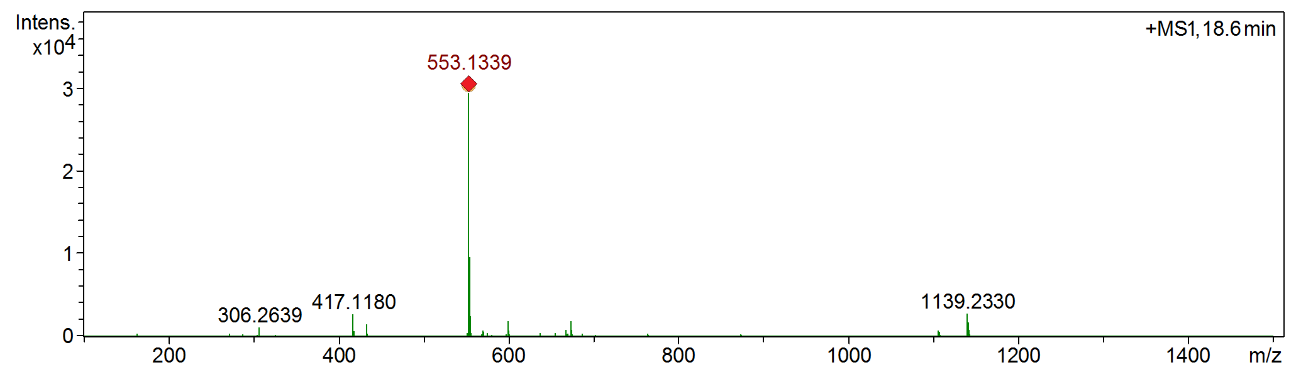

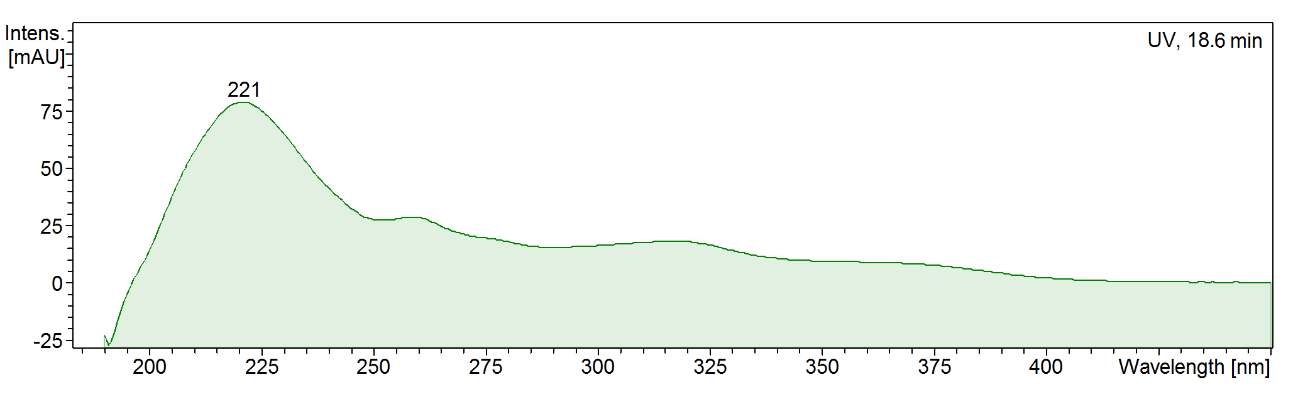


Figure 78S. MS^2^ spectrum of C-glycosyl xanthone present in the ethanolic extract of *F. formosa* leaves

Figure 77S. MS^1^ spectrum of C-glycosyl xanthone present in the ethanolic extract of *F. formosa* leaves

Figure 76S. UV spectrum of C-glycosyl xanthone present in the ethanolic extract of *F. formosa* leaves
